# Multimodal Investigations of Structural and Functional Brain Alterations in Anorexia and Bulimia Nervosa and Their Relationships to Psychopathology

**DOI:** 10.1016/j.biopsych.2024.11.008

**Published:** 2024-11-23

**Authors:** Xinyang Yu, Lauren Robinson, Marina Bobou, Zuo Zhang, Tobias Banaschewski, Gareth J. Barker, Arun L.W. Bokde, Herta Flor, Antoine Grigis, Hugh Garavan, Penny Gowland, Andreas Heinz, Rüdiger Brühl, Jean-Luc Martinot, Marie-Laure Paillère Martinot, Eric Artiges, Frauke Nees, Dimitri Papadopoulos Orfanos, Hervé Lemaître, Luise Poustka, Sarah Hohmann, Nathalie Holz, Christian Bäuchl, Michael N. Smolka, Argyris Stringaris, Henrik Walter, Robert Whelan, Julia Sinclair, Gunter Schumann, Ulrike Schmidt, Sylvane Desrivières

**Affiliations:** Social, Genetic and Developmental Psychiatry Centre, Institute of Psychiatry, Psychology and Neuroscience, King’s College London, London, United Kingdom; Department of Psychological Medicine, Centre for Research in Eating and Weight Disorders, Institute of Psychiatry, Psychology and Neuroscience, King’s College London, London, United Kingdom; South London and Maudsley NHS Foundation Trust, London, United Kingdom; Oxford Institute of Clinical Psychology Training and Research, Oxford University, Oxford, United Kingdom; Social, Genetic and Developmental Psychiatry Centre, Institute of Psychiatry, Psychology and Neuroscience, King’s College London, London, United Kingdom; Division of Psychiatry and Department of Clinical, Educational & Health Psychology, University College London, London, United Kingdom; Social, Genetic and Developmental Psychiatry Centre, Institute of Psychiatry, Psychology and Neuroscience, King’s College London, London, United Kingdom; Department of Child and Adolescent Psychiatry and Psychotherapy, Central Institute of Mental Health, Medical Faculty Mannheim, Heidelberg University, Mannheim, Germany; Department of Neuroimaging, Institute of Psychiatry, Psychology and Neuroscience, King’s College London, London, United Kingdom; Discipline of Psychiatry, School of Medicine, Dublin and Trinity College Institute of Neuroscience, Trinity College Dublin, Dublin, Ireland; Institute of Cognitive and Clinical Neuroscience, Central Institute of Mental Health, Medical Faculty Mannheim, Heidelberg University, Mannheim, Germany; Department of Psychology, School of Social Sciences, University of Mannheim, Mannheim, Germany; NeuroSpin, Commissariat à l’Energie Atomique, Université Paris-Saclay, Gif-sur-Yvette, France; Departments of Psychiatry and Psychology, University of Vermont, Burlington, Vermont; Sir Peter Mansfield Imaging Centre School of Physics and Astronomy, University of Nottingham, Nottingham, United Kingdom; Department of Psychiatry and Psychotherapy CCM, Charité – Universitätsmedizin Berlin, Freie Universität Berlin, Humboldt, Universität zu Berlin, and Berlin Institute of Health, Berlin, Germany; Physikalisch-Technische Bundesanstalt, Braunschweig and Berlin, Germany; Institut National de la Santé et de la Recherche Médicale, Institut National de la Santé et de la Recherche Médical U1299 “Developmental trajectories & psychiatry”, Université Paris-Saclay, Université Paris Cité, Centre National de la Recherche Scientifique, Centre Borelli UMR9010, Gifsur-Yvette, France; Institut National de la Santé et de la Recherche Médicale, Institut National de la Santé et de la Recherche Médical U1299 “Developmental trajectories & psychiatry”, Université Paris-Saclay, Université Paris Cité, Centre National de la Recherche Scientifique, Centre Borelli UMR9010, Gifsur-Yvette, France; AP-HP, Sorbonne Université, Department of Child and Adolescent Psychiatry, Pitié-Salpêtrière Hospital, Paris, France; Institut National de la Santé et de la Recherche Médicale, Institut National de la Santé et de la Recherche Médical U1299 “Developmental trajectories & psychiatry”, Université Paris-Saclay, Université Paris Cité, Centre National de la Recherche Scientifique, Centre Borelli UMR9010, Gifsur-Yvette, France; Psychiatry Department, EPS Barthélémy Durand, Étampes, France; Department of Child and Adolescent Psychiatry and Psychotherapy, Central Institute of Mental Health, Medical Faculty Mannheim, Heidelberg University, Mannheim, Germany; Institute of Cognitive and Clinical Neuroscience, Central Institute of Mental Health, Medical Faculty Mannheim, Heidelberg University, Mannheim, Germany; Institute of Medical Psychology and Medical Sociology, University Medical Center Schleswig Holstein, Kiel University, Kiel, Germany; NeuroSpin, Commissariat à l’Energie Atomique, Université Paris-Saclay, Gif-sur-Yvette, France; NeuroSpin, Commissariat à l’Energie Atomique, Université Paris-Saclay, Gif-sur-Yvette, France; Institut des Maladies Neurodégénératives, UMR 5293, Centre National de la Recherche Scientifique, Commissariat à l’Energie Atomique, Université de Bordeaux, Bordeaux, France; Department of Child and Adolescent Psychiatry and Psychotherapy, University Medical Centre Göttingen, Göttingen, Germany; Department of Child and Adolescent Psychiatry, Psychotherapy and Psychosomatics, University Medical Center Hamburg-Eppendorf, Hamburg, Germany; Department of Child and Adolescent Psychiatry and Psychotherapy, Central Institute of Mental Health, Medical Faculty Mannheim, Heidelberg University, Mannheim, Germany; Faculty of Psychology, Technische Universität Dresden, Dresden, Germany; Department of Psychiatry, Technische Universität Dresden, Dresden, Germany; Department of Psychiatry and Neuroimaging Center, Technische Universität Dresden, Dresden, Germany; Division of Psychiatry and Department of Clinical, Educational & Health Psychology, University College London, London, United Kingdom; Department of Psychiatry and Psychotherapy CCM, Charité – Universitätsmedizin Berlin, Freie Universität Berlin, Humboldt, Universität zu Berlin, and Berlin Institute of Health, Berlin, Germany; School of Psychology and Global Brain Health Institute, Trinity College Dublin, Dublin, Ireland; Clinical and Experimental Sciences, Faculty of Medicine, University of Southampton, Southampton, United Kingdom; Centre for Population Neuroscience and Stratified Medicine, Department of Psychiatry and Neuroscience, Charité – Universitätsmedizin Berlin, Berlin, Germany; Centre for Population Neuroscience and Precision Medicine, Institute for Science and Technology of Brain-inspired Intelligence, Fudan University, Shanghai, China; Department of Psychological Medicine, Centre for Research in Eating and Weight Disorders, Institute of Psychiatry, Psychology and Neuroscience, King’s College London, London, United Kingdom; South London and Maudsley NHS Foundation Trust, London, United Kingdom; Social, Genetic and Developmental Psychiatry Centre, Institute of Psychiatry, Psychology and Neuroscience, King’s College London, London, United Kingdom

## Abstract

**Background:**

Neurobiological understanding of eating disorders (EDs) is limited. This study presents the first comparative multimodal magnetic resonance imaging assessments of anorexia nervosa and bulimia nervosa and uncovers neurobiological differences associated with these disorders.

**Methods:**

This case-control study included 57 healthy female control participants and 130 female participants with EDs (bulimia nervosa and anorexia nervosa subtypes). Structural and functional magnetic resonance imaging assessed gray matter volume (GMV), cortical thickness, and task-based activities related to reward processing, socioemotional functioning, and response inhibition. Whole-brain group differences were correlated with ED psychopathology.

**Results:**

Significant structural differences were observed in the ED group compared with healthy control participants, including reduced GMV in the left lateral orbitofrontal cortex and lower cortical thickness in the left rostral middle frontal gyrus and precuneus after adjusting for body mass index. Specific structural alterations were only evident in anorexia nervosa subgroups. GMV reductions in the orbitofrontal cortex were linked to impulsivity, while lower cortical thickness in the frontal gyrus correlated with cognitive restraint in eating, suggesting that these regions may play key roles in ED psychopathology. Functional magnetic resonance imaging also revealed notable differences. During reward anticipation, participants with EDs exhibited deactivations in the cerebellum and right superior frontal gyrus together with reduced activation in the left lingual gyrus. These functional changes were associated with heightened neuroticism. Mediation analyses suggested that starvation-related GMV reductions in EDs disrupt reward-related brain function, increase neuroticism, and reinforce cognitive restraint, likely contributing to the persistence of ED symptoms.

**Conclusions:**

These findings illuminate key neurobehavioral mechanisms that underlie EDs and point to potential brain-based targets for developing specialized treatment.

Eating disorders (EDs) are serious and hard-to-treat psychiatric disorders with high mortality and significant disability (1). The 2 main diagnostic subtypes are anorexia nervosa (AN)—characterized by an intense fear of weight gain or disturbed body image—and bulimia nervosa (BN)—characterized by recurrent episodes of binge eating and compensatory behaviors to prevent weight gain (2,3). Common psychological comorbidities, such as mood and anxiety disorders, contribute to adverse outcomes of EDs (4,5). Research into the neurobiological underpinnings of EDs has expanded in recent years (6,7) and holds promise for developing more effective treatments (6,8). Structural magnetic resonance imaging (sMRI) and functional MRI (fMRI) have yielded important insights into the neurobiology of EDs, although most studies have focused on AN, thereby limiting our understanding.

Meta-analyses of sMRI have demonstrated globally reduced gray matter volumes (GMVs) in people with AN compared with healthy control participants (HCs) (8–10), with the largest effects being observed in the thalamus (11). Similarly, reduced cortical thickness (CT) has been observed in AN, predominantly in the parietal and occipital lobes (11–13), with fewer affected regions in the frontal lobes (11,14). Findings in BN are conflicting. Some studies have reported lower GMVs in the inferior frontal gyrus (IFG) (15) or caudate (16), while others have found higher GMVs in the medial orbitofrontal cortex (OFC) and ventral striatum (17). This inconsistency about the anatomical location and extent of differences may reflect the paucity of studies that have investigated BN and the fact that, with the exception of one recent study that focused on AN (11), the current literature is based on studies with small sample sizes in which either GMV or CT was assessed (18).

Task-based fMRI studies suggest that alterations in brain mechanisms of reward, emotional processing, and response inhibition underlie ED behaviors and symptoms (19,20). Dys-regulations within and/or between limbic and executive frontostriatal circuits in particular have been hypothesized to contribute to extreme eating behaviors in AN and BN, together with shared comorbid traits, such as personality and anxiety (21), but limited data support this hypothesis. Despite a few studies on reward processing in EDs (22), it has been suggested that an imbalance between reward and inhibition characterizes these disorders (20). Compared with HCs, individuals with AN typically show an enhanced ability to delay rewards (23) and low reward reactivity (24), which may contribute to the maintenance of persistent food restriction (25,26). Conversely, BN involves deficits in frontostriatal control circuits, which may lead to diminished inhibitory control (21,27,28) and affect reward-based learning (29). However, most studies have focused on AN rather than on BN or comparisons between AN and BN, and almost none have used multimodal data combining sMRI and fMRI in relation to EDs. The focus on predefined regions of interest (ROIs) also restricts our knowledge of ED neurobiology.

To address these limitations, here we provide comprehensive, whole-brain characterizations of structural and functional brain alterations in EDs. Our MRI measures include GMV, CT, and blood oxygen level–dependent responses during reward, social-emotional processes, and response inhibition tasks. We performed between-group analyses to distinguish brain signatures that characterize ED and its subtypes and explored how these brain differences correlated with ED psychopathology.

## Methods and Materials

### Participants

Data were acquired from a female case-control cohort comprising 57 HCs and participants who met the diagnostic criteria for AN or BN (*n* = 65/group) according to DSM-5 (2) and the Eating Disorder Diagnostic Scale (30). The clinical sample was recruited through the Eating Disorders Unit at the South London and Maudsley National Health Service Foundation Trust or via social media for the ESTRA or STRATIFY studies. HCs were recruited during the third follow-up of the IMAGEN study (31). All participants were ages 18 to 25 years and of European ancestry and were recruited in London (Supplemental Methods). The ESTRA, STRATIFY, and IMAGEN studies used identical study procedures to ensure group comparability. Written informed consent was obtained from all participants before their participation.

### Psychopathology Assessments

Personality traits were assessed using the revised NEO Personality Inventory (32) and Substance Use Risk Profile Scale (33); ED behaviors were measured using the short version of the Three-Factor Eating Questionnaire (34,35), focusing on cognitive restraint (CR), emotional eating (EE), and uncontrolled eating (UE). Comorbid symptoms, including depressive symptoms, anxiety symptoms, and harmful drinking, were examined using the Patient Health Questionnaire-9 (36), the anxiety section from the Development and Well-Being Assessment (37), and the Alcohol Use Disorders Identification Test (38), respectively (Supplemental Methods).

### MRI Acquisition and Preprocessing

The acquisition and preprocessing of sMRI and fMRI data are detailed in Supplemental Methods.

### Structural MRI

Our analysis primarily targeted GMV and CT alterations because GMV reductions are significant and replicable abnormalities in AN (39), and CT is considered biologically informative and particularly sensitive to structural changes in AN (11,40). To ensure a comprehensive comparison between clinical samples and HCs, we also examined group differences in other surface-based measures (Supplemental Methods and Supplemental Results).

### Functional MRI

The analysis incorporated 3 fMRI paradigms relevant to reinforcement-related behaviors (41) crucial to understanding EDs: the monetary incentive delay (MID) task, emotional face task (EFT), and stop signal task (SST) (Supplemental Methods). In the MID task, we contrasted brain activation during the anticipation of a large win versus anticipation of no win (i.e., reward anticipation) and between feedback of a large win and no win (i.e., reward feedback). For the EFT, we contrasted brain activation during viewing of angry faces versus control stimuli. In the SST, contrasts were chosen between brain activation during a successful stop and a successful go (i.e., successful inhibition) and between a failed stop and a successful go (i.e., unsuccessful inhibition).

### Statistical Analyses

To analyze differences in demographics, body mass index (BMI), personality traits, ED behaviors, and comorbid symptoms between individuals with EDs (AN or BN) and HCs, we conducted a 1-way analysis of covariance, adjusting for age. The analysis was performed using R version 4.1.0, with *p* values corrected for multiple comparisons using the Holm-Bonferroni method.

#### Whole-Brain Analyses

Whole-brain analyses used generalized linear models in SPM12 to explore differences in sMRI and fMRI across groups, adjusting for age and scanners. In sMRI, whole-brain vertexwise morphometry analyses were used to examine anatomical differences in voxel- or surface-based measures, including total intracranial volume as an additional covariate for volumetric comparisons. BMI was considered as a covariate to account for its potential influence on structural changes when indicated in the results. In fMRI, whole-brain analyses assessed neural response variations between groups. To address multiple comparisons, cluster-wise familywise error correction was applied at *p* < .05, with a height cluster-forming threshold of *p* < .001 across the whole brain. Significant clusters identified in the statistical difference maps were neuroanatomically located using the Automated Anatomical Labeling 3 atlas (for GMV) or the Desikan-Killiany atlas (for CT). Sensitivity analyses were performed to assess the effects of BMI and comorbid symptoms on brain structural alterations (Supplemental Methods).

#### ROI-Based Analyses

Brain regions that demonstrated significant group differences in the whole-brain analyses were selected as ROIs to further investigate their associations with ED-related psychopathology. *p* Values were adjusted for multiple comparisons using the false discovery rate method.

#### Mediation Analyses

Reasoning that behavior and personality constructs emerge in response to biological processes that occur in the brain, mediation models were performed to investigate whether ED-related psychopathology mediated the relationships between brain measures and ED diagnoses (AN or BN status). Continuous variables were standardized to *z* scores. Confidence intervals for the mediation effect were estimated using the PROCESS macro for R (version 4.1.0) and based on 5000 bootstrap samples. PROCESS model 4 was used for simple mediation models, and model 6 was used for the serial mediation model.

## Results

### Sample Characteristics

Our analysis included 187 participants (65 AN, including 23 restrictive subtype [AN-R] and 42 binge eating/purging subtype [AN-BP], 65 BN, and 57 HC), excluding some participants with incomplete neuroimaging data from specific MRI analyses (Table 1). As expected, the AN group had a significantly lower BMI than the BN and HC groups. They were also slightly younger than those in the HC group (AN = 21.70 ± 2.08 years, HC = 22.63 ± 0.62 years, *p* = .01), but there were no age differences between the AN and BN or BN and HC groups. Participants in the BN group scored higher on EE and UE than participants in the HC and AN groups; participants in the AN and BN groups had higher CR than HCs. There were also differences in personality traits and comorbid symptoms; neuroticism, hopelessness, depression, and anxiety symptoms were significantly higher in the AN and BN groups than in the HC group. The BN group had higher impulsivity and more harmful drinking than the other groups (*p*s < .001).

### Neuroanatomical Correlates of EDs

Whole-brain voxelwise analyses were run to identify GMV and CT differences between the ED groups.

#### Gray Matter Volume

**Figure 1:**
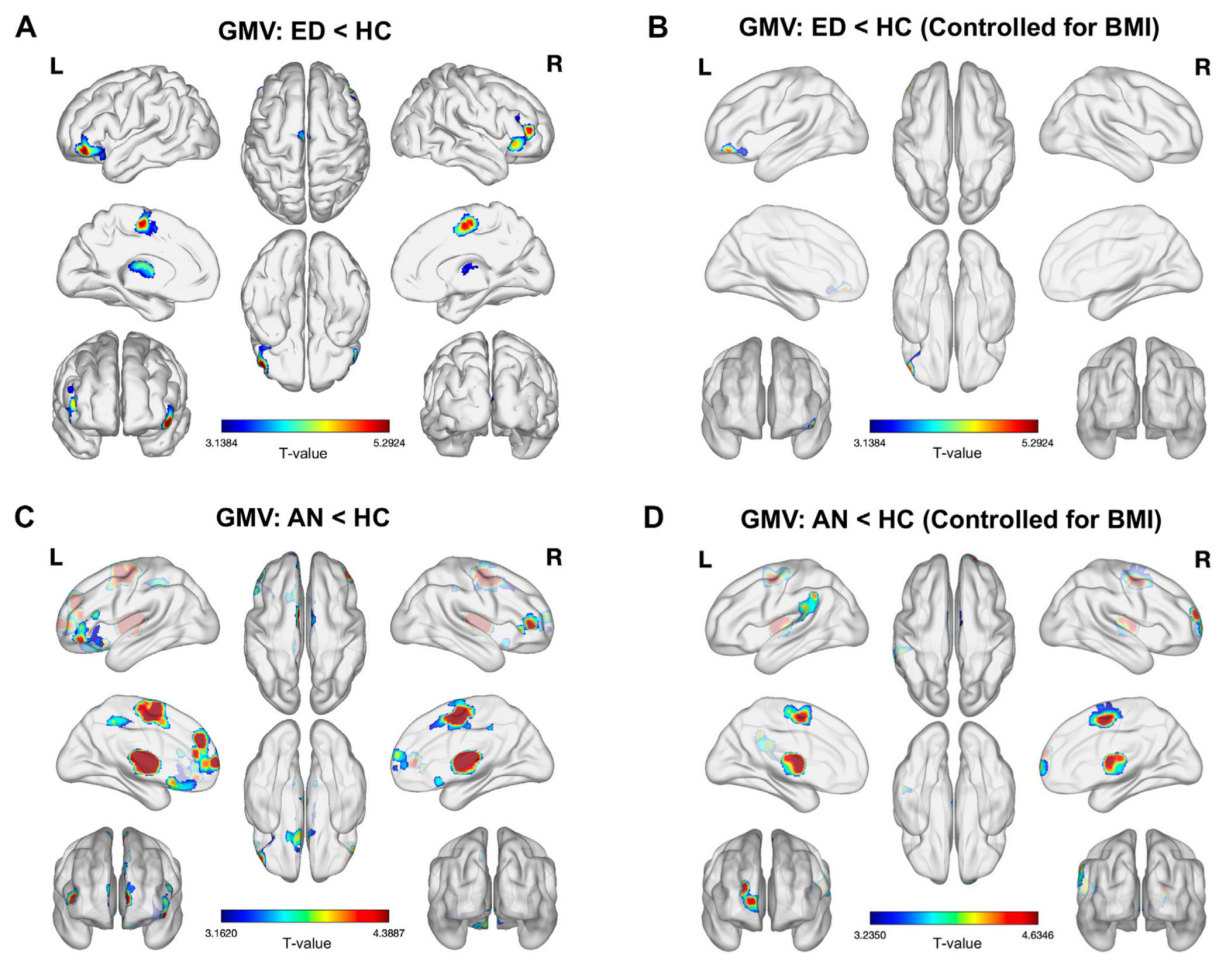
Regional differences in gray matter volume (GMV) between participants with an eating disorder (ED) (anorexia nervosa [AN] and bulimia nervosa) and healthy control participants (HCs). **(A)** Brain regions showing significant differences in whole-brain analyses when comparing the ED group to the HC group. **(B)** GMV differences between ED and HC groups, adjusted for body mass index (BMI). **(C)** GMV differences between the AN and HC groups. **(D)** GMV differences between the AN and HC groups, adjusted for BMI. No volumetric differences were found between the AN and bulimia nervosa groups when we controlled for BMI, and no differences were found between the bulimia nervosa and HC groups. The images illustrate views from the left (L) and right (R) brain hemispheres, the top rows being lateral, the middle medial, and the bottom anterior and posterior. The middle column displays superior and inferior views of the brain. The color bar indicates T values. All analyses were adjusted for age, scanning site, and total intracranial volume. A cluster-level familywise error–corrected *p* < .05 was used as the significance threshold for all comparisons.

Compared with HCs, participants with EDs exhibited significantly lower GMVs in 4 clusters, with peaks in the bilateral supplementary motor area (SMA); right middle frontal gyrus (MFG) (extending to the IFG pars orbitalis [IFGorb]); left thalamus; and left IFGorb/posterior orbital gyrus (Figure 1A and Table S1). After controlling for BMI, only the cluster in the left IFGorb/lateral OFC remained significant (Figure 1B), suggesting that it is unlikely that GMV differences in this region were due to BMI effects. Analyses of the AN and BN groups separately indicated that most GMV differences in the ED group were driven by the AN group (Figure 1C and Table S1). In the AN group, GMV differences in the SMA and thalamus remained significant after controlling for BMI (Figure 1D). Additionally, smaller GMVs in the left inferior parietal lobule/supramarginal gyrus and right medial superior frontal gyrus (SFG) were significant.

#### Cortical Thickness

**Figure 2:**
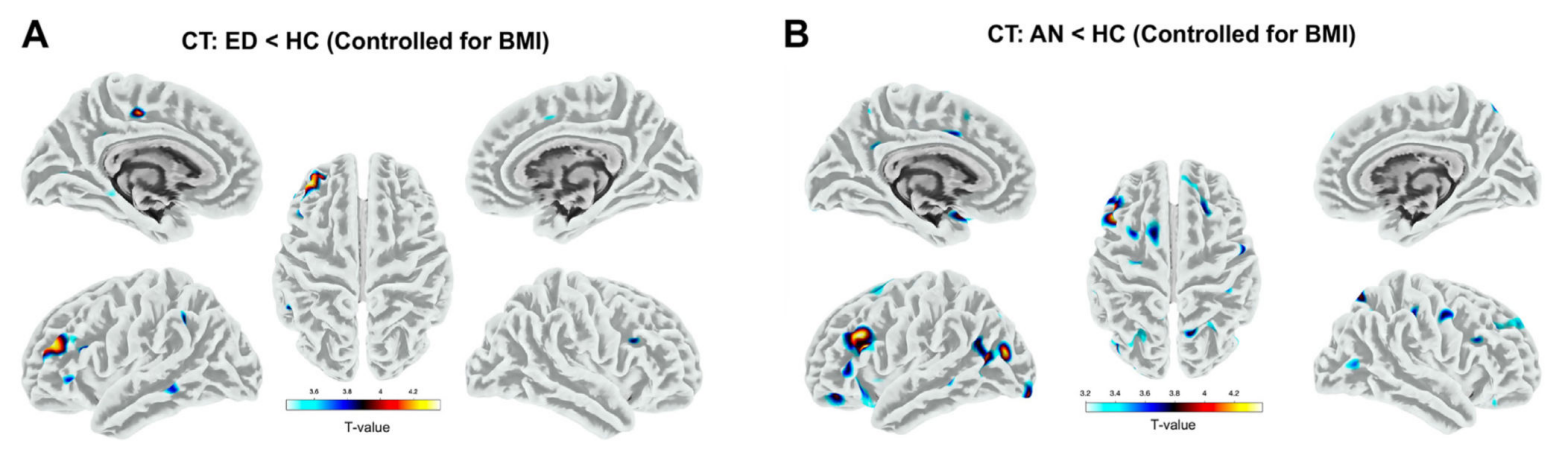
Regional differences in cortical thickness (CT) between participants with an eating disorder (ED) (anorexia nervosa [AN] and bulimia nervosa) and healthy control participants (HCs) when controlling for body mass index (BMI). **(A)** Brain regions with significant differences in whole-brain analyses when comparing the ED and HC groups. **(B)** CT differences between the AN and HC groups. The color bar indicates T values. All analyses adjusted for age and scanning site. A cluster-level familywise error–corrected *p* < .05 was used as the significance threshold for all comparisons.

Participants with EDs exhibited smaller thickness in several left-lateralized regions than HCs, including the left rostral MFG, paracentral lobule, lingual gyrus/precuneus, and left middle temporal gyrus (Table S2). When BMI was controlled for (Figure 2A), CT differences in the left rostral MFG and left precuneus remained significant. Here again, CT differences were largely driven by the AN group, with lower CT in 10 clusters identified in the AN group (compared with HCs), including in the left rostral MFG (Figure 2B).

Additional analyses of AN subgroups characterized by restrictive (AN-R) or binge-purge (AN-BP) behaviors are reported in Supplemental Results and Tables S1 and S2. Sensitivity analyses indicated that the observed neuroanatomical correlates were not driven by age, BMI, or other outliers (Supplemental Results and Table S3).

### Structural Alterations in EDs and Their Relationships to Eating Behaviors and Personality

**Figure 3:**
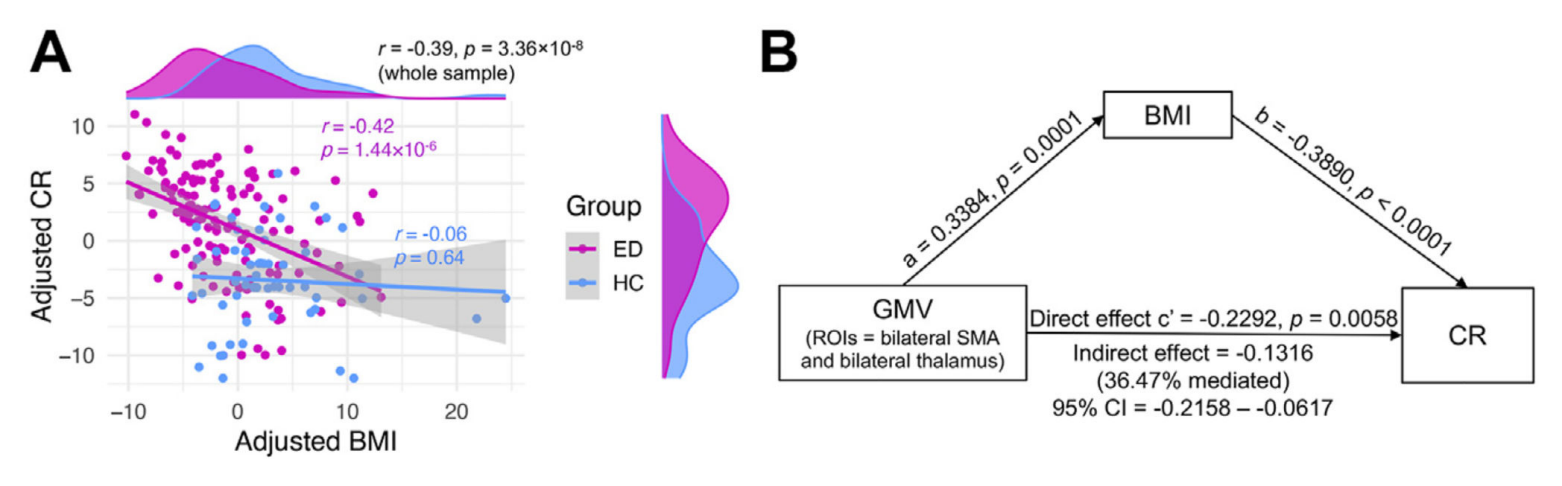
**(A)** Correlation between cognitive restraint (CR) and body mass index (BMI). The analysis was adjusted for age and recruitment site. **(B)** Mediation analyses examining relationships between lower gray matter volumes (GMVs) in the bilateral supplementary motor area (SMA) and bilateral thalamus (regions that differentiated the anorexia nervosa group from the healthy control participant [HC] group), BMI, and CR. These analyses indicated that BMI partially mediated the relationships between GMV changes and CR. ED, eating disorder; ROI, region of interest.

Next, we investigated whether the neuroanatomical differences identified above correlated with eating behaviors (CR, EE, UE), personality, and comorbid symptoms in the total sample. Using brain clusters that distinguished participants in the ED or AN group from HCs in whole-brain analyses as ROIs and adjusting for BMI (Table S4), we found that GMVs in the left lateral OFC/IFGorb (differentiating EDs from HCs) were not correlated with eating behaviors but rather with impulsivity (*r* = −0.24). GMV in ROIs associated with CR differentiated participants in the AN group from HCs, notably in the SMA (*r* = −0.26) and thalamus (*r* = −0.26). Thickness of the left rostral MFG (differentiating ED and AN from HCs) was also negatively associated with CR (*r* = −0.26, all *p*s_Bonferroni_ < .05). CR negatively correlated with BMI in the ED group (Figure 3A), and BMI partially mediated the relationship between GMV differences in the SMA and thalamus (i.e., AN-related regions) and CR (36.47% mediation) (Figure 3B). The direct relationship (*c*′ = −0.23, *p* = 5.8 × 10^−3^) between GMV in these regions and CR still remained, independently of BMI. In contrast, BMI did not mediate the relationship between thickness in the left rostral MFG and CR. These results suggest a role for the left rostral MFG, SMA, and left thalamus in the etiology of EDs via their effect on CR.

### Functional Alterations in EDs

**Figure 4:**
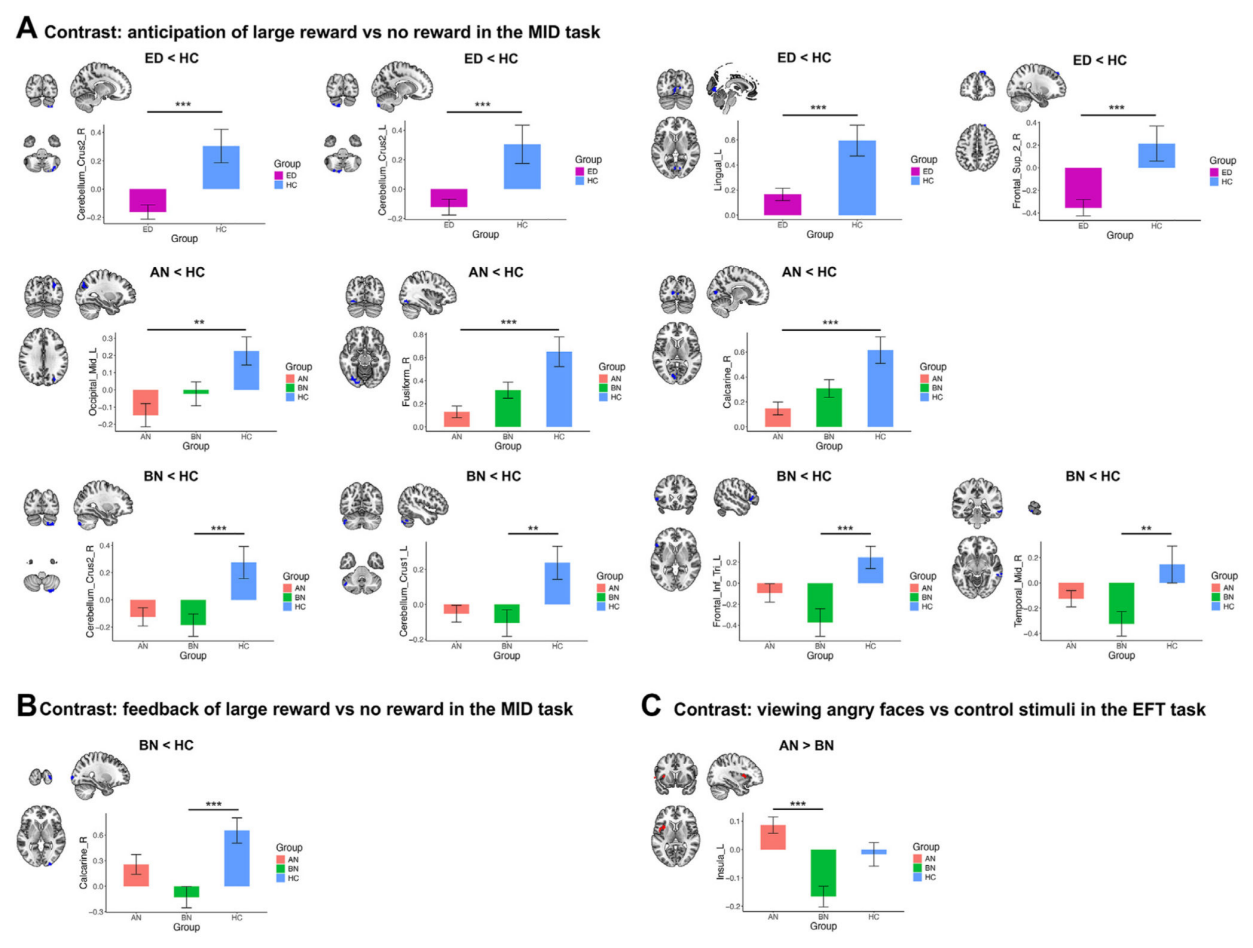
Group differences in brain activity between participants with an eating disorder (ED) (anorexia nervosa [AN] and bulimia nervosa [BN]) and healthy control participants (HCs) in 3 functional magnetic resonance imaging tasks. A cluster-level familywise error–corrected *p* < .05 was used as the significance threshold for all comparisons. **(A)** Differences in brain activation during the reward anticipation phase (anticipation of a large reward vs. anticipation of no reward) in the monetary incentive delay (MID) task when comparing the ED and the HC group (top panel), the AN and the HC group (middle panel), and the BN and the HC group (bottom panel). No differences were found between the AN and BN groups. **(B)** Group differences between the BN and HC groups during the reward feedback phase (feedback of large reward vs. no reward) of the MID task. No other differences between groups were found in this contrast. (C) AN vs. BN group differences in the left insula when viewing angry faces vs. control stimuli in the emotional face task (EFT). No other group differences were found in this task. No differences were found among these groups in the stop signal task. All analyses were adjusted for age and scanning site. The error bars indicate standard error. **p < .01, ***p < .001. L, left; R, right.

Whole-brain fMRI analyses were conducted to investigate group differences in brain activation patterns related to reward (MID task), socioemotional processing (EFT), and response inhibition (SST) (Figure 4 and Tables S5 and S6).

#### MID Task

During reward anticipation (Figure 4A and Table S5), ED group participants showed deactivations in the bilateral cerebellum (crus II) and right SFG compared with HCs and lower activations in the visual cortex (left lingual gyrus/right calcarine fissure). Comparing the AN and BN groups with the HC group revealed that differences in cerebellar activations were driven by the BN group, while lower activations in the left lingual gyrus/right calcarine fissure were driven by the AN group. In addition, deactivations in the right middle temporal gyrus and the triangular part of the left IFG were also observed in the BN and AN groups, with lower activations or deactivation in other visual areas (right fusiform gyrus and left middle occipital gyrus [MOG]). Lower activation in the left MOG, along with areas in the right frontal cortex, were also found to be associated with AN-R (Supplemental Results and Table S6). During reward feedback, no differences were found when we compared ED group participants to HCs, although BN group participants had significantly lower activations in the right calcarine fissure/superior occipital gyrus than HCs (Figure 4B and Table S5).

#### Emotional Face Task

No differences in brain activations were observed between ED group participants and HCs when they viewed angry faces versus control stimuli. The only significant differences in this task were observed when comparing the AN group to the BN group (Table S5). When viewing angry faces, participants with AN tended to activate the left insula, while participants with BN showed deactivation in this region (Figure 4C).

#### Stop Signal Task

No significant group differences were found between the ED, AN, or BN groups and the HC group. Analyses of AN subgroups revealed differences in AN-R and AN-BP compared with HCs (Supplemental Results and Table S6).

The observed alterations were not driven by age, BMI, or other outliers (Supplemental Results and Table S7).

### Relationships Between Task-Based Brain Activation Patterns in EDs, Eating Behaviors, and Personality

Analyses in the total sample were conducted to explore relationships between altered brain activations in EDs, eating behaviors, personality, and comorbid symptoms using brain clusters that differentiated ED groups in the fMRI tasks as ROIs. The only brain activations significantly associated with these traits were those identified in the MID task (Table S8). During reward anticipation, activations in ED-related ROIs were specifically associated with CR, but not EE or UE, most significantly in the left lingual/right calcarine fissure (*r* = −0.29). As for associations with personality, activations during reward anticipation in all ROIs that distinguished participants in the ED group from HCs (i.e., the cerebellum, left lingual gyrus/right calcarine fissure, and right SFG) negatively correlated with neuroticism, but not with impulsivity. They were also nominally associated with depression. In contrast, activations in the triangular part of the left IFG (i.e., ROI deactivated during reward anticipation in BN) negatively correlated with impulsivity (*r* = −0.30, all *p*s_Bonferroni_ < .05).

**Figure 5:**
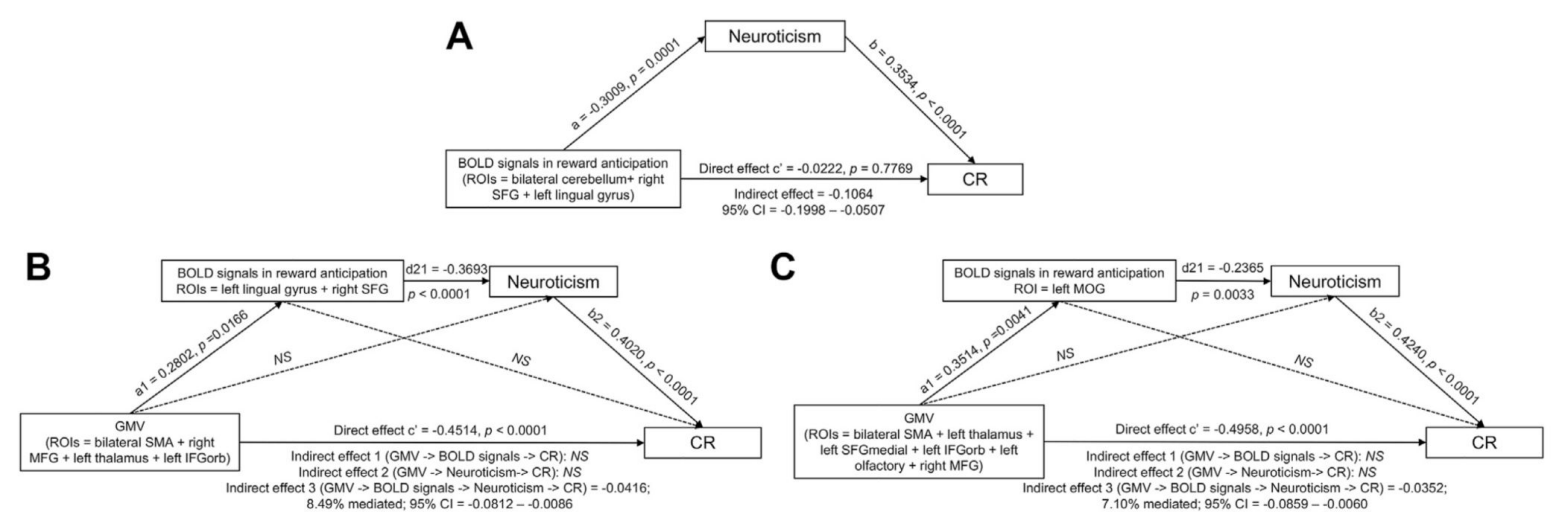
**(A)** Neuroticism as mediator of the relationship between brain deactivations during reward anticipation in regions of interest (ROIs) differentiating participants with an eating disorder from healthy control participants (HCs) and cognitive restraint (CR). **(B)** Serial mediation analyses in which brain activation patterns distinguishing participants with an eating disorder from HCs (i.e., in the left lingual gyrus and right superior frontal gyrus [SFG]) and neuroticism are tested as mediators of the relationship between gray matter volume (GMV) in ROIs differentiating participants with an eating disorder from HCs and CR. (C) Serial mediation analyses investigating brain activation patterns distinguishing participants with anorexia nervosa from HCs (i.e., in the left middle occipital gyrus [MOG]) and neuroticism as mediators of the relationship between GMV in ROIs differentiating participants with anorexia nervosa from HCs and CR. Analyses were conducted in the total sample. The model in **(A)** controlled for age, scanning site, and body mass index. The models in **(B)** and **(C)** adjusted for age, scanning site, and total intracranial volume and were no longer significant when considering the effect of body mass index. BOLD, blood oxygen level–dependent; IFGorb, inferior frontal gyrus, pars orbitalis; MFG, middle frontal gyrus; NS, not significant; SMA, supplementary motor area.

Mediation analyses revealed that neuroticism fully mediated the relationship between brain activations during reward anticipation in ED-related ROIs (i.e., the bilateral cerebellum, left lingual gyrus, and right SFG) and CR. This mediation was significant when controlling for BMI (Figure 5A).

### Relationships Between Structural Alterations, Brain Activations During Reward Anticipation, Neuroticism, and CR

Finally, we investigated whether the relationships between brain responses during reward anticipation, neuroticism, and CR might be related to structural brain alterations associated with EDs. For this, we first summed the GMVs or CTs within ROIs defined by brain regions that differed between groups (i.e., ED vs. HC or AN vs. HC) and investigated their associations with brain activation patterns within ROIs that differentiated between groups in the total sample. Nominally significant findings were found for GMV but not CT (Table S9). These correlations were only observed for ROIs derived from voxel-wise whole-brain analyses that were not corrected for BMI. GMV in regions that distinguished ED group participants from HCs (i.e., SMA, right MFG, left thalamus, left IFGorb) correlated with brain activations in the left lingual gyrus/right calcarine fissure and right SFG (both ROIs, *r* = 0.17, *p* < .05). GMVs in regions that distinguished the AN group from the HC group (SMA, left thalamus, left medial SFG, left IFGorb, left olfactory, and right MFG) correlated with brain activations in the left MOG (*r* = 0.28, *p* = .006).

Next, we investigated whether lower GMV in these ED- or AN-related brain regions might be related to brain responses during reward anticipation, neuroticism, and CR in 2 serial mediation models wherein brain activations during reward anticipation and neuroticism mediated the relationship between GMV and CR. The model using ED-related ROIs (Figure 5B) was significant: brain activations in the left lingual gyrus/right calcarine fissure and right SFG, together with neuroticism, partially mediated (8.5% mediation) the relationship between GMV in ED-related ROIs and CR. The model using AN-related ROIs (Figure 5C) was also significant, indicating that activity of the left MOG and neuroticism mediated (7.1% mediation) the relationship between GMV in AN-related ROIs and CR. In both models, the relationships between GMV and anticipatory brain activations in the MID were dependent on BMI because the mediating effects were lost after controlling for BMI. This suggests that in ED, lower GMV induced by starvation/low weight influences anticipatory brain responses to rewards and thereby influences neuroticism and CR.

## Discussion

This study represents the first multimodal, whole-brain MRI investigation comparing ED participants—including AN subtypes (AN-R and AN-BP) and BN—to HCs, highlighting structural and functional brain alterations associated with EDs and their relationships to psychopathological traits. After controlling for BMI, participants in the ED group exhibited reduced GMV in the left lateral OFC/IFGorb, which was linked to increased impulsivity, and reduced CT in the left rostral MFG, which was associated with CR in eating. When analyzed separately, specific structural differences were observed only in the AN subgroups. fMRI analyses revealed disrupted anticipatory brain responses to rewards in EDs characterized by deactivations in the cerebellum (driven by the BN group) and the right SFG, as well as decreased activation in the left lingual gyrus (driven by the AN group). These functional alterations correlated with heightened neuroticism, which fully mediated the relationship between altered reward responses in these regions and CR. Serial mediation analyses further demonstrated that BMI-related GMV differences influenced reward anticipation, contributing to elevated neuroticism and CR in EDs. These findings elucidate key neurobehavioral mechanisms that underlie EDs and offer a promising foundation for the development of targeted, brain-based interventions that are aimed at addressing specific neurocognitive impairments associated with impulsivity, CR, and reward anticipation.

Our analyses comparing participants with ED to HCs while controlling for the impact of BMI on brain structure (42) suggest that the lateral OFC/IFGorb and rostral MFG play roles in the pathophysiology of EDs rather than being the consequence of low weight or starvation. The finding that lower GMV in the OFC correlated with impulsivity is consistent with studies that have shown that patients with damage to this region are more impulsive (43). The lateral OFC, which is functionally connected with the IFG (44), is involved in flexible decision making by associating sensory stimuli with predicted outcomes (45,46). In relation to eating behaviors, studies suggest that the OFC assigns reward values to food, thereby guiding behavioral choices (47). For example, in rodent studies, activation of feeding-responsive neurons in the OFC causes increased feeding behavior (48), which may contribute to weight gain and obesity by biasing consumption toward highly palatable and rewarding foods.

Regarding the left rostral MFG, reduced thickness in this region negatively correlated with CR independently of BMI. This region is associated with craving regulation for food and substances such as nicotine (49,50). Interestingly, food cravings are more closely associated with mood and EE than food deprivation and CR (51). The lower thickness in the left rostral MFG in EDs, notably in the AN-BP subtype, may thus underlie difficulties in regulating craving, potentially leading to the need for greater CR, the conscious restriction of food intake to control body weight and shape.

Our study also expands existing knowledge by identifying structural alterations in fronto-temporo-occipital areas that distinguish AN subtypes from HCs. Notably, in addition to the alterations in the left rostral MFG noted above, lower volumes in the thalamus, which is a hub for brain function and connectivity in BN (52), and parietal regions (parietal lobule and left supramarginal gyrus) were specific to AN-BP. In contrast, lower volumetric alterations in frontal regions (SMA and SFG), which integrate sensory and reward information (53,54), and fusiform and lingual gyri, which are involved in visual information processing and abnormal body image perception (55,56), were specific to AN-R. The volumes of the SMA and left thalamus, lower in the AN group, were associated with CR even after controlling for the effect of BMI, suggesting a role for these brain regions in the etiology of EDs. Consistent with our expectations (57), as CR increased, BMI decreased. Accordingly, CR was particularly pronounced in AN, possibly due to pronounced avoidance of food driven by intense fear of weight gain and a preoccupation with body shape, leading to low BMI. However, given the 2 dimensions of dietary restraint (58), CR may also create food cravings that may lead to binge eating or overeating in some individuals (e.g., AN-BP). Thus, the identified links between SMA volume and AN-R and thalamus volume and AN-R raise the interesting possibility that these 2 brain regions may be related to distinct aspects of CR.

Functionally, the disrupted anticipatory brain responses to rewards in the cerebellum observed in EDs, notably BN, support a prominent role for this region in feeding behavior. Lack of cerebellar activation in anticipatory responses to food has been associated with hyperphagia, and disruption of a cerebellum-driven satiety network controlling striatal dopamine release has been proposed as an underlying mechanism (59). Our finding of lower cerebellar activation in the ED/BN groups supports the cerebellum’s role in reducing the reward value of food intake upon satiety and contributing to overeating. Alternatively, the bilateral crus I/II deactivations seen during reward anticipation in BN may reflect deficits in working memory (60). In contrast, disrupted anticipatory brain responses in the lingual gyrus, characterized by disrupted activation patterns in occipitotemporal visual areas, were driven by AN. These regions are activated by visual craving-inducing cues such as drugs (61), and activation of the lingual gyrus has been inversely related to food craving in individuals with obesity, possibly reflecting increased attentional or visual processing efforts to reduce craving (62).

Our findings suggest that alterations in GMVs in brain regions structurally affected by EDs influence CR, partly through their impact on anticipatory brain activations and neuroticism. This relationship is both novel and intriguing. Notably, neuroticism fully mediated the relationship between anticipatory brain activations in ED-related regions and CR independently of BMI. However, the influence of GMV differences on CR, through brain activation and neuroticism, was related to BMI. This suggests that in EDs, GMV alterations caused by starvation or low weight disrupt reward-related brain function and heighten neuroticism, thereby reinforcing CR. This mechanism likely contributes to the maintenance of ED symptoms. These findings support a cognitive-behavioral theory of AN, which posits that once the disorder begins, a feedback loop is triggered wherein starvation reinforces further dietary restriction, making the disorder self-perpetuating (63).

While our multimodal investigation into EDs offers valuable insights, several limitations should be acknowledged. First, our study included only female participants of White ethnicity, limiting the generalizability of the findings to populations with more gender and ethnic diversity. Second, the age range of participants was relatively narrow, which confines the applicability of our results to the earlier stages of EDs despite these disorders often having a prolonged course. Although we controlled for age in our analyses, significant age differences between the HCs and participants with EDs were noted. Future studies should aim for closer age matching to reduce potential confounding effects. Third, the lack of randomization in the task order may have influenced our results because negative affective processes could have impaired subsequent cognitive performance (64). Fourth, while mediation analyses were used to explore relationships between brain structure, personality traits, and ED behaviors, the cross-sectional nature of our data means that these findings should be interpreted with caution and without inferring causality. Finally, our study did not account for potential confounding variables such as illness duration, medication or treatment status, and socioeconomic factors, all of which should be considered when interpreting our results.

## Conclusions

Our study identified critical neurobehavioral mechanisms that underlie EDs, shedding light on both shared and distinct features across different ED subtypes. Specific structural and functional alterations observed in ED subgroups provide a more nuanced understanding of the complexity of these disorders. Key brain regions, such as the left OFC, rostral MFG, cerebellum, and left lingual gyrus, were found to play significant roles in ED pathology. These findings highlight potential novel brain-based targets for developing specialized and effective interventions for EDs.

## Supplementary Material

Supplementary material cited in this article is available online at https://doi.org/10.1016/j.biopsych.2024.11.008.

Supplementary

## Figures and Tables

**Table 1:** Participant Characteristics Stratified by Analyses

	HC	AN	BN	*F*	*P*				Post Hoc Analyses				
						AN vs. HC			BN vs. HC			AN vs. BN	
						*P*	95% CI		*P*	95% CI		*P*	95% CI
Whole Sample													
*N*	57	65	65	−	−	−	−		−	−		−	−
Age, Years,Mean (SD) [Range]	22.63 (0.62) [21.98−24.79]	21.70 (2.08) [18.11−26.19]	22.21 (2.01) [18.74−28.00]	*F*_2,184_ = 4.42	.01	.01	−1.69 to −0.17		.54	−1.18 to 0.33		.27	−1.23 to 0.21
BMI, Mean (SD) [Range]	25.24 (5.46) [18.35−45.96]	16.92 (2.83) [13.06−21.92]	24.06 (3.87) [18.79−36.06]	*F*_2_,_183_ = 70.17	2.40 × 10^−13^	1.04 × 10^−20^	−10.14 to −6.41		.37	−2.99 to 0.66		1.11 × 10^−17^	−8.88 to −5.34
Eating Behaviors—TFEQ													
Cognitive restraint	14.58 (4.23)	22.43 (3.56)	18.75 (4.47)	*F*_2_,_183_ = 52.78	7.98 × 10^−19^	2.31 × 10^−19^	6.00 to 9.70		2.59 × 10^−7^	2.37 to 5.98		3.00 × 10^−6^	1.92 to 5.44
Emotional eating	6.79 (2.58)	5.98 (2.66)	9.94 (2.13)	*F*_2,183_ = 48.60	1.18 × 10^−17^	.08	−2.11 to 0.09		2.24 × 10^−10^	2.00 to 4.15		5.41 × 10^−17^	−5.13 to −3.04
Uncontrolled eating	19.93 (5.85)	19.72 (6.26)	27.08 (5.41)	*F*_2,183_ = 33.63	3.64 × 10^−13^	**>**.999	−3.22 to 2.01		1.23 × 10^−9^	4.44 to 9.56		1.68 × 10^−11^	−10.10 to −5.11
Personality —NEO-PI-R													
Neuroticism	1.84 (0.58)	2.99 (0.62)	2.85 (0.65)	*F*_2,183_ = 55.97	1.08 × 10^−19^	1.68 × 10^−17^	0.82 to 1.37		1.87 × 10^−15^	0.72 to 1.26		**>**.999	−0.16 to 0.37
Extraversion	2.46 (0.57)	1.98 (0.66)	2.29 (0.61)	*F*_2_,_183_ = 9.07	1.76 × 10−4	1.49 × 10^−4^	−0.75 to −0.20		.45	−0.43 to 0.11		.013	−0.58 to 0.05
Openness	2.45 (0.57)	2.51 (0.54)	2.80 (0.56)	*F*_2,183_ = 7.54	7.11 × 10−4	**>**.999	−0.25 to 0.25		3.78 × 10^−3^	0.08 to 0.57		2.74 × 10^−3^	−0.56 to −0.09
Agreeableness	2.87 (0.44)	2.48 (0.50)	2.56 (0.51)	*F*_2,183_ = 8.71	2.44 × 10−4	3.82 × 10^−4^	−0.57 to −0.14		3.28 × 10^−3^	−0.51 to −0.08		**>**.999	−0.27 to 0.15
Conscientiousness	2.70 (0.52)	2.48 (0.69)	2.25 (0.63)	*F*_2_,_183_ = 7.78	5.70 × 10−4	.51	−0.43 to 0.12		4.73 × 10^−4^	−0.70 to −0.16		.04	0.01 to 0.53
Personality—SURPS													
Hopelessness	1.79 (0.38)	2.91 (0.63)	2.45 (0.63)	*F*_2_,_183_ = 53.74	4.36 × 10^−19^	1.61 × 10^−19^	0.83 to 1.34		7.23 × 10^−9^	0.40 to 0.90		5.90 × 10^−5^	0.20 to 0.68
Anxiety sensitivity	2.37 (0.42)	2.62 (0.45)	2.70 (0.56)	*F*_2_,_183_ = 7.27	9.20 × 10^−4^	.03	0.02 to 0.45		7.10 × 10^−4^	0.12 to 0.54		0.77	−0.3 to 0.11
Impulsivity	1.90 (0.39)	2.14 (0.53)	2.48 (0.54)	*F*_2_,_183_ = 20.69	7.59 × 10^−9^	.08	−0.02 to 0.43		6.74 × 10^−9^	0.35 to 0.78		1.81 × 10^−4^	−0.57 to −0.15
Sensation seeking	2.64 (0.54)	2.67 (0.65)	2.87 (0.60)	*F*_2_,_183_ = 3.20	.04	**>**.999	−0.30 to 0.23		.02	−0.05 to 0.47		.06	−0.50 to 0.01
Comorbid Symptoms													
Depression	3.43 (3.58)	15.22 (5.90)	14.19 (6.48)	*F*_2_,_183_ = 75.78	2.14 × 10^−24^	2.57 × 10^−21^	8.92 to 13.93		1.55 × 10^−19^	8.18 to 13.14		**>**.999	−1.68 to 3.21
Anxiety, DAWBA	1.27 (0.56)	2.68 (1.23)	2.63 (1.26)	*F*_2_,_183_ = 30.51	4.28 × 10^−12^	2.97 × 10^−10^	0.91 to 1.90		7.14 × 10^−10^	0.87 to 1.84		**>**.999	−0.43 to 0.53
Harmful drinking	5.40 (3.07)	5.74 (5.68)	9.40 (7.57)	*F*_2_,_183_ = 8.27	3.67 × 10^−4^	**>**.999	−2.84 to 2.65		4.13 × 10^−3^	0.95 to 6.46		9.89 × 10^−4^	−6.31 to −1.29
Sample for sMRI Analyses (Including GMV and CT)													
*n*	56	64	57	−	−	−	−		−	−		−	−
Age, Years	22.72 (0.62)	21.78 (2.10)	22.53 (2.09)	*F*_2,174_ = 4.76	.01	−	−		−	−		−	−
BMI	24.91 (4.72)	16.62 (1.80)	24.06 (4.00)	*F*_2_,_173_ = 96.32	1.11 × 10^−28^	−	−		−	−		−	−
Total Intracranial Volume, mm^3^	1397.96 (94.17)	1416.80 (105.01)	1415.61 (113.76)	*F*_2,173_ = 0.59	.558	−	−		−	−		−	−
Sample for fMRI Analyses: MID Task													
*n*	49	48	53	−	−	−	−		−	−		−	−
Age, Years	22.67 (0.59)	21.75 (2.09)	22.51 (2.07)	*F*_2,147_ = 3.92	.02	−	−		−	−		−	−
BMI	24.90 (4.60)	16.58 (1.62)	24.27 (3.95)	*F*_2,146_ = 79.05	4.91 × 10^−24^	−	−		−	−		−	−
Sample for fMRI Analyses: EFT													
*n*	54	62	61	−	−	−	−		−	−		−	−
Age, Years	22.72 (0.63)	21.79 (2.10)	22.48 (2.10)	*F*_2,174_ = 4.30	.02	−	−		−	−		−	−
BMI	24.78 (4.68)	16.71 (1.76)	24.17 (3.97)	*F*_2,173_ = 91.88	5.81 × 10^−28^	−	−		−	−		−	−
Sample for fMRI Analyses: SST													
*n*	50	61	61	−	−	−	−		−	−		−	−
Age, Years	22.73 (0.64)	21.92 (2.06)	22.49 (2.09)	*F*_2,169_ = 3.07	.05	−	−		−	−		−	−
BMI	24.94 (4.77)	16.56 (1.79)	24.09 (3.94)	*F*_2,168_ = 93.82	3.92 × 10^−28^	−	−		−	−		−	−

Values are presented as mean (SD) unless otherwise indicated. The comparisons of age were controlled for sites. BMI, eating behaviors, personality, and comorbid symptoms were controlled for age and site. AN, anorexia nervosa; BMI, body mass index; BN, bulimia nervosa; CT, cortical thickness; DAWBA, Development and Well-Being Assessment; EFT, emotional face task; fMRI, functional magnetic resonance imaging; GMV, gray matter volume; HC, healthy control participant; MID, monetary incentive delay; NEO-PI-R, Revised NEO Personality Inventory; sMRI, structural MRI; SST, stop signal task; SURPS, Substance Use Risk Profile Scale; TFEQ, Three-Factor Eating Questionnaire.
