# Supplementary material for: Multimodal Investigations of Structural and Functional Brain Alterations in Anorexia and Bulimia Nervosa and Their Relationships to Psychopathology
Source: Biol Psychiatry. Author manuscript; Available in PMC 2026 Apr 16. (PMC7619009; doi:10.1016/j.biopsych.2024.11.008)
Supplement: Supplementary [file EMS213148-supplement-Supplementary.docx]

**SUPPLEMENTARY INFORMATION**

**Multimodal Investigations of Structural and Functional Brain Alterations in Anorexia and Bulimia Nervosa and Their Relationships to Psychopathology**

Yu *et al.*

[Supplementary methods 3](#_Toc178664236)

[Participants 3](#_Toc178664237)

[Psychopathological assessments 4](#_Toc178664238)

[MRI data acquisition and preprocessing 6](#_Toc178664239)

[fMRI paradigms 9](#_Toc178664240)

[Structural measures 11](#_Toc178664241)

[Statistical analyses 11](#_Toc178664242)

[Supplementary results 13](#_Toc178664243)

[Psychiatric comorbidities in clinical groups 13](#_Toc178664244)

[Intelligence and neurocognitive performance across the AN, BN, and HC groups 13](#_Toc178664245)

[Structural alterations associated with BMI or comorbid symptoms 13](#_Toc178664246)

[Whole-brain functional alterations associated with personality traits 14](#_Toc178664247)

[Sub-analysis: Structural and functional differences between AN subtypes, BN and HC 15](#_Toc178664248)

[Sensitivity analysis 1: Exclusion of participants with AUD diagnosis 17](#_Toc178664249)

[Sensitivity analysis 2: Comparing participants acquired with different MRI scanners 18](#_Toc178664250)

[Sensitivity analysis 3: Effect of age 18](#_Toc178664251)

[Sensitivity analysis 4: Inclusion of HCs within the healthy weight range only 18](#_Toc178664252)

[No group differences in other surface-based measures 18](#_Toc178664253)

[Supplementary Figures 19](#_Toc178664254)

[Figure S1. Volumetric differences between different AN subtypes and their comparisons with HC, after adjusting for BMI effect. No significant differences were found between AN subtypes and BN. 19](#_Toc178664255)

[Figure S2. Differences in cortical thickness between different AN subtypes and their comparisons with HC, after controlled for BMI. No differences in cortical thickness were found between AN subtypes and BN after BMI adjustment. 20](#_Toc178664256)

[Figure S3. Distributions of age (a) and BMI (b) in the AN, BN, and HC groups from this study. For age, a sensitivity analysis was conducted excluding ED participants under 22 years old and the BN participant aged 28 years to address the potential developmental differences. For BMI, we excluded HCs with a BMI over 25 or below 18.5 to include only participants of healthy weight in our sensitivity analysis. 21](#_Toc178664257)

[Supplementary Tables 22](#_Toc178664258)

[Table S1. Whole-brain voxel-wise analyses of gray matter volume (GMV) identified brain clusters exhibiting significant differences between groups. 23](#_Toc178664259)

[Table S2. Whole-brain voxel-wise analyses of cortical thickness (CT) identified brain clusters exhibiting significant differences between groups. 25](#_Toc178664260)

[Table S3. Sensitivity analyses for structural alterations. 27](#_Toc178664261)

[Table S4. Relationships between neuroanatomical correlates of EDs (i.e., GMV and CT) and eating behaviours, comorbid symptoms & personality: whole sample analysis. 30](#_Toc178664262)

[Table S5. Whole-brain voxel-wise analyses of fMRI tasks (the MID, EFT, and SST) identified brain clusters exhibiting significant differences in brain activations between groups. 32](#_Toc178664263)

[Table S6. Whole-brain voxel-wise analyses of fMRI tasks identified brain clusters exhibiting significant functional differences between the two AN subtypes (AN-R and AN-BP) and their differences from BN and HC groups. 34](#_Toc178664264)

[Table S7. Sensitivity analyses of functional alterations. 36](#_Toc178664265)

[Table S8. ROIs-based analyses of fMRI BOLD signals identified through whole-brain voxel-wise analysis, and their associations with eating behaviors, personality, and comorbid symptoms in the whole sample. 38](#_Toc178664266)

[Table S9. Correlations between GMVs/CT of regions that differed between groups and the brain activations in the regions identified from whole-brain fMRI analysis. 39](#_Toc178664267)

[Table S10. Differences in intelligence and cognitive function between groups. 40](#_Toc178664268)

# Supplementary methods

## Participants

All participants, including patients and healthy controls, completed the Patient Health Questionnaire-9 (1) that was used for diagnosis of major depressive disorder (MDD); the Alcohol Use Disorders Identification Test (2), for alcohol use disorder (AUD), F20-F29 ICD-10 for psychosis (3); and the Eating Disorder Diagnostic Scale (4), for DSM-5-based (5) diagnoses of AN or BN. All cohorts used in our study followed the same study protocols, including questionnaire and MRI assessments and were scanned on the same scanners, ensuring their comparability.

*Clinical sample:* Participants with AN (*N* = 59) and BN (*N* = 43) were recruited from the Eating Disorder Unit at the South London and Maudsley NHS Foundation Trust as part of the ESTRA study (https://estrastudy.co.uk/). To enlarge the sample size and investigate comorbidity, we included participants from a sister study that recruited participants with MDD and AUD (STRATIFY; (https://stratify-project.org/). This resulted in the further inclusion of AN or BN participants with initial MDD (PHQ-9 ≥ 15; *N* = 11); or AUD diagnosis (AUDIT total score ≥ 15; *N* = 16). The final sample included 65 AN and 65 BN participants.

Diagnoses and associated comorbidities in the clinical sample

| **Cohort** | **AN** | **BN** |
| --- | --- | --- |
| **ESTRA** | **59 (all met AN criteria)**  34 (57.6%) also met the criteria for MDD  2 (3.4%) also met the criteria for AUD | **43 (all met BN criteria)**  21 (48.8%) also met the criteria for MDD  7 (16.3%) also met the criteria for AUD |
| **STRATIFY** | **6 (all met AN criteria)**  3 MDD (50%)  3 AUD (50%) | **22 (all met BN criteria)**  8 MDD (36.36%)  13 AUD (59.09%)  1 Psychosis (4.5%) |
| **Total N** | **65 (all met AN criteria)**  37 MDD (56.9%)  5 AUD (7.7%) | **65 (all met BN criteria)**  29 MDD (44.6%)  20 AUD (30.8%)  1 Psychosis (1.5%) |

AN participants were further categorized as outlined in the DSM-5(5) and EDDS as binge-eating and purging subtype of AN (AN-BP; i.e., those who engaged in binge eating, purging, or a combination of both behaviors in the past three months) and restricting AN (AN-R; i.e., those who reported no regular binge eating and/or purging behaviors). Among the 65 AN participants, 23 were classified as AN-R and 42 as AN-BP.

Fifty-seven healthy controls (HC) were recruited as part of the IMAGEN study in the third follow-up cohort. The IMAGEN study is a large-scale community-based longitudinal imaging-genomic study of brain development, which employed standardized methods for multicenter acquisition of neuroimaging, behavioral, and neuropsychological assessment (6). HCs were screened negative for all mental illness diagnoses based on the Mini-International Neuropsychiatric Interview version 5.0 (7) during the institute assessment.

Both the clinical and HC groups were comprised of female individuals, aged 18-25 years, of white ethnicity, and recruited in London. Participants were excluded from the study if they had brain injuries or neurobiological disorders; severe hearing or vision difficulties; type I or type II diabetes, or were heavily medicated for serious illness other than for the mental health issue under investigation; were pregnant; or had restricted mobility that would prevent them from lying flat for 1.5 hours during the MRI scan.

## Psychopathological assessments

*Personality traits*: We used the Revised NEO Personality Inventory (NEO-PI-R) and Substance Use Risk Profile Scale (SURPS) to measure the personality traits of all participants. The NEO-PI-R contains 60 self-reported items based on five subscales of neuroticism, extraversion, openness, agreeableness, and conscientiousness (8). The 23-item self-reported SURPS was used to assess the levels of several personality risk factors, including hopelessness, anxiety sensitivity, impulsivity, and sensation seeking, that may predispose individuals to substance use and other mental disorders (9).

*ED behaviors*: The short version of the Three-Factor Eating Questionnaire (TFEQ) was used to assess disordered eating behaviors. The questionnaire consists of three subscales, namely: cognitive restraint, which refers to the tendency to restrict one’s food intake constantly and consciously instead of using physiological cues, hunger, and satiety, as regulators of food intake; emotional eating, which pertains to the tendency to eat in response to negative emotions; and uncontrolled eating, which describes the tendency to overeat while experiencing a sense of loss of control. The questionnaire has demonstrated good psychometric properties and has been widely used and validated across European populations (10,11).

*Other mental health symptoms*: depressive symptoms were measured using the Patient Health Questionnaire-9 (PHQ-9), a 9-item sub-scale designed to screen for depressive disorders (1). Each item evaluates the presence of one of the nine criteria outlined in the DSM-IV for depressive disorder during the last two weeks. The total PHQ-9 score was computed to evaluate the severity of depressive symptoms. Anxiety symptoms were assessed through the band score obtained from the anxiety section (section G) of the Development and Well-Being Assessment (DAWBA), a diagnostic tool designed to generate psychiatric diagnoses following the ICD-10 and DSM-IV criteria (12). The total score of the Alcohol Use Disorders Identification Test (AUDIT) was used to assess harmful drinking (2).

*Intelligence*: We used the short form of the Wechsler Adult Intelligence Scale (WAIS; Pearson plc, London, UK) to measure the intelligence of both cases and controls. The average scores of the verbal comprehension and perceptual reasoning subscales were used to assess participants’ intelligence levels.

*Neurocognitive tests*: To evaluate the cognitive functions of our participants, we administered the Cambridge Gambling Task (CGT), the Intra-Extra Dimensional Set Shift (IED), and the Spatial Working Memory (SWM) tests from the Cambridge Neuropsychological Test Automated Battery (CANTAB)(13). Briefly, the CGT assesses decision-making and risk-taking behavior outside a learning context, measuring risk-taking, quality of decision-making, decision time, risk adjustment, delay aversion, and impulsivity. The IED tests rule acquisition and reversal, assessing visual discrimination, attention maintenance, and flexibility. The outcome measures assess the number of errors made, the number of trials completed, the number of stages completed, and latency. The SWM evaluates participants’ ability to retain spatial information and to manipulate remembered items in working memory, and the outcome measures include errors and strategy.

## MRI data acquisition and preprocessing

Structural and functional MRI data were acquired using 3T MRI scanners from Siemens and General Electric. Scanning parameters were specifically chosen to be compatible across both scanners, and the same scanning protocol was used at both scanning sites. In brief, high-resolution T1-weighted three-dimensional structural images were acquired for anatomical localization and co-registration with the functional time series. Blood oxygen level-dependent (BOLD) functional images were acquired with a gradient-echo, echo-planar imaging sequence. For all tasks, 300 volumes were acquired for each participant, and each volume consisted of 40 slices aligned to the anterior commission/posterior commission line (2.4-mm slice thickness, 1 mm gap). The echo time was optimized (echo time = 30 ms; repetition time = 2,200 ms) to provide reliable imaging of subcortical areas.

*Preprocessing of structural MRI data*: Prior to preprocessing, all raw structural images were visually inspected to identify and discard any affected by movement artifacts, brace artifacts, or field inhomogeneities. Subsequent preprocessing was conducted using the Computational Anatomy Toolbox (CAT 12.6 r1450; <https://neuro-jena.github.io/cat/>) in SPM 12 (Wellcome Department of Cognitive Neurology) to obtain grey matter volumes (GMVs), cortical thickness (CT), and other surface-based measures, such as sulcal depth, gyrification and cortical complexity. Our primary focus was on GMV and CT alterations, as reductions in GMV have been identified as among the most prominent and replicable abnormalities in AN (14), and CT has been hypothesized to be a more biologically informative measure with particular sensitivity to structural changes, especially in AN (15,16). However, to ensure a comprehensive comparison between the clinical samples and healthy controls, we also examined group differences in other surface-based measures. T1-weighted structural images were segmented into grey matter (GM), white matter (WM), and cerebrospinal fluid (CSF), and then transformed into the standard Montreal Neurological Institute (MNI) space using the DARTEL algorithm. To obtain GMVs, the GM voxel values were modulated by the Jacobian determinant obtained from the spatial normalization step. Total intracranial volumes (TIV) were estimated by adding up GM, WM, and CSF. All normalized GM images were finally smoothed with an isotropic Gaussian kernel of 8mm full width at half maximum (FWHM) for GMV analyses, surface area data were resampled and smoothed with 15 mm for cortical thickness and sulcal depth, and 20 mm for folding measures, respectively. Quality assessment reports were generated and reviewed during preprocessing, with only images of or above sufficient quality (corresponding to grade D) being included in subsequent analyses.

*Functional MRI preprocessing*: Functional imaging data were preprocessed and analyzed using SPM12 (Statistical Parametric Mapping; [www.fil.ion.ucl.ac.uk/spm](http://www.fil.ion.ucl.ac.uk/spm)). The spatial preprocessing involved several steps: slice time correction to adjust for time differences resulting from multi-slice imaging acquisition; realignment to the first volume in line; nonlinear warping to the Montreal Neurological Institute (MNI) space using a custom EPI template derived from the average of the mean images of 400 adolescents; resampling to a voxel size of 3 × 3 × 3 mm^3^; and smoothing with a 5-mm FWHM isotropic Gaussian kernel. Specifically, all fMRI data underwent visual inspection by trained lab technicians using AFNI’s standard display (with axial, sagittal, and coronal views). Technicians visually inspected images for signs of anatomical abnormalities, motion artifacts, scanner artifacts, and overall completeness of the scans. The fMRI images were then overlaid onto a standard template in MNI space to check the quality of coregistration and normalization. Then, using the standard 6-parameter (3 translations, 3 rotations) motion estimates with volume 0 as the reference, the maximum and minimum displacement (in any direction) was identified per subject per task. Participants exceeding the standard criteria of 1 voxel dimension (+/- 3 mm) can easily be identified for further inquiry. Likewise, framewise displacement (FD) was calculated based on Power et al., 2012 (17). The overall mean FD, along with the associated volume index where FD ≥ 0.5, was calculated per subject per task. Participants with a mean FD ≥ 0.5 were identified and excluded. Volumes exceeding FD ≥ 0.5 were identified and censored. No global signal regression was performed in the preprocessing of the fMRI data in the current study.

At the first level of analysis, changes in the BOLD response for each subject were assessed by linear combinations of experimental conditions at the individual subject level. For each experimental condition, each trial was convolved with the hemodynamic response function to form regressors that accounted for potential noise variance (e.g., head movement) associated with the corresponding experimental condition. Estimated movement parameters were added to the design matrix in the form of 18 additional columns (three translations, three rotations, three quadratic translations, and three cubic translations, plus a shift of ± 1 TR (repetition time) for each translation).

## fMRI paradigms

*Stop signal task (SST):* Participants performed an event-related adapted version of the SST to examine neural responses to successful and unsuccessful inhibitory control (18). The task was composed of 480 Go trials and 80 unpredicted Stop trials. During Go trials, participants were instructed to respond to an arrow pointing either left or right by pressing the corresponding button with their left or right index finger. In Stop trials, an upward arrow was presented after an average delay of 300 ms following the initial arrow, and participants were instructed to inhibit their motor responses during these trials. A tracking algorithm was used to change the time interval between the Go signal and the Stop signal onsets according to each participant’s performance on previous trials (average percentage of inhibition over precious Stop trials, recalculated after each Stop trial), resulting in 50% successful and 50% unsuccessful inhibition trials to ensure that participants worked at the edge of their own inhibitory capacity. The inter-trial interval was 1800 ms. The Go Success condition was modeled explicitly to improve estimation accuracy and as a baseline. The chosen contrasts of interest were successful stop vs. successful go (indicating successful inhibitory control) and failed stop vs. successful go (indicating unsuccessful inhibitory control).

*Monetary incentive delay (MID) task*: Participants were subjected to a modified version of the MID task to assess neural responses to reward anticipation and reward outcome (19). The task consisted of 66 10-s trials. In each trial, participants were presented with sequences of cues (250ms, in one of three shapes), targets, and feedback. The target, a white square, would appear on the left or right side of the screen after the cue and indicate whether no reward (0 points), a small reward (2 points), or a large reward (10 points) could be won in that trial. Followed by a variable delay (4000-4500 ms) of fixation on a white crosshair, participants were instructed to respond with a left or right button press as soon as the target appeared. Feedback on whether and how many points were won during the trial was presented for 1450 ms after the response. Using a tracking algorithm, the task difficulty was individually adjusted (i.e., target duration varied between 100 and 300 ms), ensuring that each participant successfully responded in ~66% of trials. The chosen contrast of interest was large win > no win for the anticipation and feedback phases. Only trials in which participants successfully hit the target before it disappeared were included in further analyses.

*Emotional face task (EFT)*: The EFT was used to investigate emotional reactivity to social stimuli (20). During the task, all participants were instructed to watch 18-s blocks of either a face movie or a control stimulus. The face movie consisted of black and white video clips that lasted between 200 and 500 ms and were either angry or neutral (greyscale clips of male or female faces). The control stimuli included black and white concentric circles expanding the contracting at various speeds that roughly matched the contrast and motion characteristics of the face clips. Five blocks of angry and neutral expressions were interleaved with nine blocks of the control stimulus and each block contained eight trials of six face identities (three males and three females). To avoid the emotional ambiguity in the neutral faces suggested by previous studies (21), we only concentrated on the contrast of angry faces vs. control stimuli, which could be interpreted solely as the effect of viewing negative social stimuli (angry faces).

## Structural measures

*Other surface-based measures*: In addition to CT, the CAT12 toolbox also allowed us to derive other surface-based measures, such as sulcal depth, gyrification, and cortical complexity. Our main focus was on the changes in CT because it has been hypothesized to be a more biologically informative measure with particular sensitivity to structural changes, notably in AN (15,16). Nevertheless, to ensure a comprehensive comparison between the clinical samples and healthy controls, we also examined group differences in other surface-based measures.

## Statistical analyses

Whole-brain group-level fMRI analyses were conducted specifically in the following contrasts: differences in brain activation during the following contrasts: (1) anticipation of a large win versus anticipation of no win in the MID task; (2) feedback of a large win versus feedback of no win in the MID task; (3) viewing angry face versus control stimuli in the EFT; (4) successful stop versus successful go in the SST; (5) unsuccessful stop versus successful go in the SST.

The primary analysis was conducted between ED (including AN and BN) and HC groups, combining participants from two recruitment sites (i.e., scanners) and including those with comorbidities to enhance the statistical power for investigating structural and functional differences among the three groups. Age and scanner (coded as dummy variables) were included as covariates in all analyses, with TIV also controlled for in analyses involving GMV.

To further characterize our clinical participants and to gain a more comprehensive understanding of how different EDs and their clinical subtypes differentiate from HC, we conducted a sub-analysis in which AN participants were subdivided into AN-R and AN-BP to explore differences within AN, as well as between AN subtypes, BN and HC groups.

To assess the potential confounding effects of comorbidity, different scanners, age, and extreme BMI effects, we extracted the brain clusters identified from the primary whole-brain analyses as regions of interest (ROI), and conducted the following sensitivity analyses: (1) We excluded participants who met the clinical diagnostic criteria for AUD and re-run sMRI and fMRI analyses, calculated effect sizes (Cohen’s *d*) and compared them in the whole sample and the sample excluding AUD participants. (2) We compared effect sizes (Cohen’s *d*) between participants whose MRI data were acquired using the General Electric (GE) (71.1% of participants) or Siemens scanner, to address the potential effects of using different MRI scanners. (3) Given the age differences between AN and HC (AN = 21.70 ± 2.08 years, HC = 22.63 ± 0.62 years, *p* = 0.01; Fig. S3) and the potential risk that normal developmental differences may be attributed to eating disorder pathology, we excluded ED participants under 22 years old and one BN participant who was 28 years old, re-run analyses, and compared the effect sizes obtained with those of the whole sample. (4) Although BMI was not an inclusion criterion for HC, noticing that some HC participants exhibited underweight or obese BMI status (**Fig. S3**), we re-run analyses after excluding HC participants outside of a healthy weight range (as defined by a BMI between 18.5 and 24.9, based on the NHS guidelines: https://www.nhs.uk/conditions/obesity) and compared the effect sizes obtained to those derived from the whole sample.

For all sensitivity analyses, Cohen’s *d* was derived from the between-group comparison (i.e., the mean differences between the two comparison groups divided by the pooled standard deviation).

# Supplementary results

## Psychiatric comorbidities in clinical groups

Using the cutoff criteria for major depressive disorder (MDD; PHQ9 ≥ 15), alcohol use disorder (AUD; AUDIT total score ≥ 15), psychosis (based on F20-F29 ICD-10 diagnosis), out of the 65 AN participants, 37 met the criteria for MDD and 5 for AUD. Among the 65 BN participants, 29 met the criteria for MDD, 20 for AUD, and 1 for psychosis.

## Intelligence and neurocognitive performance across the AN, BN, and HC groups

Our findings showed that there were no significant differences in IQ (i.e., the average score of verbal comprehension and perceptual reasoning index scores) among the three groups (*p* > 0.05; **Table S10**). Regarding neurocognitive tests, both the AN and BN groups exhibited more risk-taking behaviors, higher impulsivity, and poorer risk adjustment in the CGT; demonstrated poorer attention shifting in the IED task; and showed poorer spatial working memory in the SWM task compared with the HC group (all *p*s < 0.05). There were no significant differences in cognitive performance between the AN and BN groups.

## Structural alterations associated with BMI or comorbid symptoms

Between-group comparisons of whole-brain voxel-wise analyses of GMV and CT were conducted to identify structural alterations in AN and BN. Given the marked relationship between low BMI and structural brain changes in AN (16), we further tested the effects of BMI and comorbidity on the associations identified. As expected, controlling for BMI had marked effects. In the GMV analyses (**Table S1**), this resulted in a decrease in the number of significant brain regions, but the largest clusters distinguishing AN from HCs (i.e., bilateral SMA and left thalamus) remained significant after controlling for BMI or comorbid anxiety and depressive symptoms. In addition, two novel clusters in the left inferior parietal gyrus (*k* = 1457, *t* = 4.33, *p*_FWE_ = 0.004) and right medial SFG (SFGmedial; cluster size *k* = 1158, *t* = 4.72, *p*_FWE_ = 0.010) became significant. In contrast, clusters that previously differentiated AN from BN (i.e., left SFGmedial and right caudate) were no longer significant.

In CT analyses (**Table S2**) controlling for BMI increased the number of significant brain clusters associated with AN. Again, the largest, located in the left rostral MFG and left IPG, remained significant after controlling for BMI or comorbid symptoms, while other clusters (i.e., left paracentral lobule, right SMG, and right precuneus) were no longer significant. New clusters in frontal, parietal, temporal and occipital areas emerged as significant (cluster peaks: SFG, *k* = 791, *t* = 4.42, *p*_FWE_ < 0.001; left inferior parietal gyrus, *k* = 516, *t* = 3.97, *p*_FWE_ < 0.001; right superior parietal gyrus, *k* = 362, *t* = 4.29, *p*_FWE_ < 0.001; right SFG, *k* = 335, *t* = 4.04, *p*_FWE_ < 0.001; left superior temporal gyrus, STG, *k* = 290, *t* = 4.09, *p*_FWE_ = 0.001; right lateral occipital gyrus, *k* = 285, *t* = 4.31, *p*_FWE_ = 0.001; right middle temporal gyrus (MTG), *k* = 165, *t* = 4.43, *p*_FWE_ = 0.027; right precentral gyrus, *k* = 157, *t* = 3.95, *p*_FWE_ = 0.033; right fusiform, *k* = 149, *t* = 4.19, *p*_FWE_ = 0.039; and right precentral gyrus, *k* = 143, *t* = 4.54, *p*_FWE_ = 0.045). The cluster in the right precuneus that differentiated AN from BN was no longer significant after BMI adjustment.

## Whole-brain functional alterations associated with personality traits

Given the observed differences in personality traits among EDs and HC groups (**Table 1**), and the potential effects of these traits on affect and emotion processing (22), we repeated our whole-brain functional analyses by additionally controlling for personality traits. During the MID task, differential activation in all brain regions identified with this task was affected by personality traits (**Table S5**). After adjusting for neuroticism, none of the observed group differences remained significant, neither during anticipation nor feedback. After controlling for hopelessness, the left superior occipital gyrus (*k* = 40; *t* = 3.97; *p_FWE_* = 0.017) remained significantly less activated in AN or BN compared with HC, during anticipation and feedback, respectively. Finally, after controlling for impulsivity, all findings comparing BN to HC lost their significance, but the clusters previously identified when comparing AN with HC (i.e., the right calcarine fissure, left middle occipital gyrus, and right fusiform gyrus) remained significant. These findings suggest that personality traits may drive reward-related brain dysfunctions in ED. During the EFT, AN showed higher brain activations in the left insula than in BN. This increased activation in the left insula maintained its significance after further controlling for personality traits in our whole-brain analyses. Additionally, controlling for neuroticism revealed a new cluster of deactivation in the right SMG (*k* = 72; *t* = 4.12; *p_FWE_* < 0.001) when comparing BN with HC.

## Sub-analysis: Structural and functional differences between AN subtypes, BN and HC

We further characterized our 65 AN participant based on their binge-eating and purging behaviors, resulting in *N* = 23 as AN-R and *N* = 42 as AN-BP subtypes. Subsequently, we examined the structural and functional differences between these subtypes and compared them with BN and HC groups (**Tables S1-2,** and **S6**).

*GMV*: After controlling for BMI, the analyses (**Table S1**) revealed that in the main analyses comparing AN to HCs, the lower GMVs in the frontal lobe (i.e., right SMA) were largely driven by the AN-R group, while the lower volumes in the bilateral thalamus were driven by the AN-BP group (**Figure S1**). Specifically, compared to HCs, AN-R showed decreased GMV in the right SMA (*k* = 1793, *t* = 4.72, *p*_FWE_ = 0.001), left SFGmedial (extending into dorsolateral superior frontal gyrus, *k* = 1382, *t* = 4.49, *p*_FWE_ = 0.004), left fusiform and lingual gyri (*k* = 1128, *t* = 5.14, *p*_FWE_ = 0.012). Compared to HCs, AN-BP had lower GMV in the bilateral thalamus (*k* = 2635, *t* = 5.56, *p*_FWE_ < 0.001), left supramarginal gyrus (*k* = 1110, *t* = 4.06, *p*_FWE_ = 0.022) and right precuneus (*k* = 967, *t* = 5.21, *p*_FWE_ = 0.039).

*CT*: When adjusting for BMI, compared to HCs, the reduced thickness in the left inferior parietal lobule, observed in AN, was also observed in both the AN-R and AN-BP groups (for AN-R: *k* = 872, *t* = 5.76, *p*_FWE_ < 0.001; for AN-BP: *k* = 348, *t* = 3.87, *p*_FWE_ < 0.001; **Table S2; Figure S2**). Group differences were found, notably in the left rostral MFG (*k* = 765, *t* = 4.90, *p*_FWE_ < 0.001), left (*k* = 460, *t* = 4.42, *p*_FWE_ < 0.001), and right superior frontal gyri (*k* = 318, *t* = 4.43, *p*_FWE_ = 0.001), which only discriminated AN-BP from HCs. Additionally, AN-BP showed lower thickness in the left postcentral gyrus (*k* = 711, *t* = 5.35, *p*_FWE_ < 0.001), left caudal middle frontal gyrus (*k* = 430, *t* = 4.46, *p*_FWE_ < 0.001), and left lateral orbitofrontal gyrus (*k* = 379, *t* = 4.28, *p*_FWE_ < 0.001). While AN-BP displayed lower thickness in the left middle temporal gyrus (*k* = 705, *t* = 4.54, *p*_FWE_ < 0.001), right paracentral gyrus (*k* = 290, *t* = 3.87, *p*_FWE_ = 0.002), left (*k* = 232, *t* = 4.65, *p*_FWE_ = 0.007) and right precuneus (*k* = 147, *t* = 4.58, *p*_FWE_ = 0.047), and left pars opercularis (*k* = 147, *t* = 3.84, *p*_FWE_ = 0.047). No significant differences were observed between AN-R or AN-BP and BN.

*MID task*: Compared to HCs, AN-R had lower activations during reward anticipation in several brain areas (**Table S6**). These included the left MOG (*k* = 32, *t* = 4.33, *p*_FWE_ = 0.035), as observed in AN, and the right frontal cortex, including the medial (*k* = 35, *t* = 4.94, *p*_FWE_ = 0.023) and medial orbital part (*k* = 38, *t* = 4.88, *p*_FWE_ = 0.015) of the right SFG, the right MFG (*k* = 73, *t* = 4.62, *p*_FWE_ < 0.001) and right SFG (*k* = 30, *t* = 4.20, *p*_FWE_ = 0.047). During the reward feedback phase, AN-R showed lower activations in the left MFG (*k* = 39, *t* = 4.78, *p*_FWE_ = 0.008). No significant differences for the other group comparisons.

*EFT*: The only significant differences found were increased activations in the left STG/rolandic operculum (*k* = 36, *t* = 4.27, *p*_FWE_ = 0.035; **Table S6**), when comparing AN-BP to BN. This activation pattern resembles the higher activation of the left insula/STG in AN compared to BN, suggesting that increased activation in this region in AN was driven by the AN-BP subgroup.

*SST*: Although we did not observe any group differences when grouping AN-R and AN-BP together, we found significant differences between the two AN subtypes and between them with HC. Specifically, when compared to HCs, AN-R showed higher activations in the left SMA (*k* = 45, *t* = 3.96, *p*_FWE_ = 0.005) and right IFG (*k* = 33, *t* = 4.65, *p*_FWE_ = 0.028), whereas AN-BP showed lower activations in the right postcentral gyrus (*k* = 38, *t* = 4.48, *p*_FWE_ = 0.012). Compared to AN-BP, AN-R showed higher activations in the right SMG (*k* = 57, *t* = 4.70, *p*_FWE_ = 0.001) and left temporal pole (*k* = 38, *t* = 5.18, *p*_FWE_ = 0.013; **Table S6**).

## Sensitivity analysis 1: Exclusion of participants with AUD diagnosis

To test if AUD influenced our primary findings, we conducted a sensitivity analysis, excluding from the ED group (AN or BN) participants who met the criteria for AUD, specifically those with a total score of 15 or higher on the Alcohol Use Disorders Identification Test (AUDIT). We then repeated the sMRI and fMRI analyses, also calculating effect sizes (Cohen’s d) for clusters identified in our primary analysis and compared them in the whole sample and the sample excluding AUD participants. For all ROIs identified in our primary analyses, effect sizes were comparable regardless of the inclusion of participants diagnosed with AUD (**Tables S3** and **S7**). This indicates that our findings were unlikely to be confounded by the presence of AUD comorbidity.

## Sensitivity analysis 2: Comparing participants acquired with different MRI scanners

We compared the effect sizes for each ROI identified in our primary whole-brain analysis between the whole sample and two subsets of the participants whose MRI data were acquired with the GE or Siemens in scanners separately. The directionality of effect sizes for participants scanned with different scanners were consistent (**Tables S3** and **S7**).

## Sensitivity analysis 3: Effect of age

Given the significant age differences between our AN and HC groups **(Table 1)** and the potential confounding effect of developmental differences, we conducted sensitivity analyses, excluding ED participants under 22 years old or above 28 years old and re-running analyses for each ROI identified in our primary whole-brain analysis. Comparisons of effect sizes obtained in the whole sample and after excluding participants show that these are generally consistent (**Tables S3** and **S7**), suggesting that our findings are not driven by developmental differences.

## Sensitivity analysis 4: Inclusion of HCs within the healthy weight range only

To address the potential confounding effects of underweight or some extremely obese participants in the HC group, we only included HC participants with a BMI between 18.5 and 24.9 and repeated our analysis (**Tables S3** and **S7**). Our results revealed that participants with extreme BMIs did not confound our conclusions from the primary analysis.

## No group differences in other surface-based measures

Using the CAT12 toolbox, we calculated and compared differences in gyrification, sulcal depth, and cortical complexity between groups with two-sample t-tests. However, no significant differences were found between groups.

# Supplementary Figures

## **Figure S1.** Volumetric differences between different AN subtypes and their comparisons with HC, after adjusting for BMI effect. No significant differences were found between AN subtypes and BN.

## Figure S2. Differences in cortical thickness between different AN subtypes and their comparisons with HC, after controlled for BMI. No differences in cortical thickness were found between AN subtypes and BN after BMI adjustment.

## Figure S3. Distributions of age (A) and BMI (B) in the AN, BN, and HC groups from this study. For age, a sensitivity analysis was conducted excluding ED participants under 22 years old and the BN participant aged 28 years to address the potential developmental differences. For BMI, we excluded HCs with a BMI over 25 or below 18.5 to include only participants of healthy weight in our sensitivity analysis.

# Supplementary Tables

| **Contents** | |
| --- | --- |
| **Table S1** | Whole-brain voxel-wise analyses of gray matter volume (GMV) identified brain clusters exhibiting significant differences between groups. |
| **Table S2** | Whole-brain voxel-wise analyses of cortical thickness (CT) identified brain clusters exhibiting significant differences between groups. |
| **Table S3** | Sensitivity analyses for structural alterations. |
| **Table S4** | Relationships between neuroanatomical correlates of EDs (i.e., GMV and CT) and eating behaviours, comorbid symptoms & personality: whole sample analysis. a, the result is significant after Bonferroni correction. |
| **Table S5** | Whole-brain voxel-wise analyses of fMRI tasks (the MID, EFT, and SST) identified brain clusters exhibiting significant differences in brain activations between groups. |
| **Table S6** | Whole-brain voxel-wise analyses of fMRI tasks identified brain clusters exhibiting significant functional differences between the two AN subtypes (AN-R and AN-BP) and their differences from BN and HC groups. |
| **Table S7** | Sensitivity analyses of functional alterations. |
| **Table S8** | ROIs-based analyses of fMRI BOLD signals, identified through whole-brain voxel-wise analysis, and their associations with eating behaviors, personality, and comorbid symptoms in the whole sample. |
| **Table S9** | Correlations between GMVs/CT of regions that differed between groups and the brain activations in the regions identified from whole-brain fMRI analysis. |
| **Table S10** | Differences in intelligence and cognitive function between groups. |

## Table S1. Whole-brain voxel-wise analyses of gray matter volume (GMV) identified brain clusters exhibiting significant differences between groups.

| Contrast | Cluster level | | | Peak level | Coordinates, MNI (mm) | | | Anatomical location (AAL3 atlas) |
| --- | --- | --- | --- | --- | --- | --- | --- | --- |
|  | *p FWE* | kE | Cohen's *d* | T | x | y | z |  |
| ***GMV*** | | | | | | | | |
| ED vs HC | 0.011 | 1244 | -0.67 | -5.13 | 2 | -12 | 57 | Right supplementary motor area |
|  |  |  |  | -5.05 | 8 | -6 | 54 |  |
|  |  |  |  | -3.84 | -2 | -8 | 70 | Left supplementary motor area |
|  | 0.014 | 1161 | -0.70 | -5.11 | 50 | 45 | 10 | Right middle frontal gyrus |
|  |  |  |  | -4.58 | 52 | 30 | -4 | Right inferior frontal gyrus, pars orbitalis |
|  |  |  |  | -3.85 | 42 | 38 | 2 |  |
|  | 0.014 | 1170 | -0.55 | -4.84 | -6 | -4 | 4 | Left thalamus, anteroventral |
|  | 0.024 | 1012 | -0.63 | -5.29 | -50 | 44 | -12 | Left inferior frontal gyrus, pars orbitalis |
|  |  |  |  | -4.31 | -52 | 34 | -12 |  |
|  |  |  |  | -3.62 | -40 | 21 | -16 | Left posterior orbital gyrus |
| AN vs HC | < 0.001 | 4685 | -0.89 | -5.48 | 0 | -12 | 57 | Left supplementary motor area |
|  |  |  |  | -5.48 | 3 | -3 | 51 | Right supplementary motor area |
|  |  |  |  | -4.97 | -2 | -6 | 72 | Left supplementary motor area |
|  | < 0.001 | 3589 | -0.77 | -6.22 | -6 | -4 | 4 | Left thalamus, anteroventral |
|  |  |  |  | -5.04 | -2 | -16 | 14 | Left thalamus, mediodorsal medial magnocellular |
|  |  |  |  | -4.77 | -15 | -10 | 15 | Left thalamus, ventral lateral |
|  | < 0.001 | 3316 | -0.81 | -5.01 | -4 | 64 | 8 | Left medial superior frontal gyrus |
|  |  |  |  | -4.91 | -4 | 45 | 28 |  |
|  |  |  |  | -4.32 | -10 | 40 | 15 | Left pregenual anterior cingulate cortex (ACC) |
|  | 0.002 | 1739 | -0.78 | -4.90 | -51 | 44 | -10 | Left inferior frontal gyrus, pars orbitalis |
|  |  |  |  | -4.66 | -54 | 32 | -10 |  |
|  |  |  |  | -3.82 | -46 | 40 | 3 |  |
|  | 0.010 | 1180 | -0.66 | -5.19 | 48 | 45 | 9 | Right middle frontal gyrus |
|  |  |  |  | -4.16 | 51 | 30 | -2 | Right inferior frontal gyrus, triangular part |
|  | 0.036 | 828 | -0.87 | -4.00 | -6 | 16 | -15 | Left olfactory cortex |
|  |  |  |  | -3.71 | -9 | 38 | -18 | Left gyrus rectus |
|  |  |  |  | -3.70 | -16 | 20 | -21 | Left medial orbital gyrus |
| AN vs BN | 0.001 | 2098 | -0.65 | -4.39 | -3 | 46 | 21 | Left medial superior frontal gyrus |
|  |  |  |  | -3.97 | -2 | 10 | 44 | Left supplementary motor area |
|  |  |  |  | -3.87 | -10 | 50 | 16 | Left medial superior frontal gyrus |
|  | 0.010 | 1167 | -0.62 | -3.91 | 24 | 18 | 10 | Right caudate |
|  |  |  |  | -3.83 | 26 | 16 | -8 | Right putamen |
|  |  |  |  | -3.68 | 14 | 6 | -4 | Right pallidum |
| ***GMV, controlled for BMI*** | | | | | | | | |
| ED vs HC | 0.003 | 1607 | -0.66 | -5.00 | -48 | 44 | -14 | Left lateral orbitofrontal cortex |
|  |  |  |  | -4.15 | -54 | 33 | -14 | Left inferior frontal gyrus, pars orbitalis |
|  |  |  |  | -3.66 | -40 | 24 | -14 |  |
| AN vs HC | < 0.001 | 2473 | -1.09 | -4.92 | 0 | -12 | 57 | Left supplementary motor area |
|  |  |  |  | -4.89 | 4 | -2 | 50 | Right supplementary motor area |
|  |  |  |  | -4.71 | -3 | -2 | 54 | Left supplementary motor area |
| AN vs HC | < 0.001 | 2296 | -0.95 | -5.32 | -6 | -4 | 4 | Left thalamus, anteroventral |
|  |  |  |  | -4.49 | -15 | -10 | 14 | Left thalamus, ventral lateral |
|  |  |  |  | -3.92 | 12 | -9 | 15 | Right thalamus, ventral lateral |
|  | 0.004 | 1457 | -1.11 | -4.33 | -56 | -45 | 36 | Left inferior parietal lobule |
|  |  |  |  | -4.19 | -57 | -34 | 22 | Left supramarginal gyrus |
|  |  |  |  | -3.82 | -62 | -46 | 28 |  |
|  | 0.010 | 1158 | -0.97 | -4.72 | 24 | 60 | 18 | Right medial superior frontal gyrus |
|  |  |  |  | -4.66 | 15 | 62 | 4 |  |
|  |  |  |  | -4.26 | 4 | 66 | 0 |  |
| AN-R vs  HC | 0.001 | 1793 | -1.37 | -4.72 | 3 | -4 | 50 | Right supplementary motor area |
|  |  |  |  | -4.00 | 6 | 12 | 46 |  |
|  |  |  |  | -3.94 | 3 | 4 | 68 |  |
|  | 0.004 | 1382 | -1.31 | -4.49 | -8 | 68 | 8 | Left medial superior frontal gyrus |
|  |  |  |  | -3.97 | -24 | 66 | 15 | Left superior frontal gyrus, dorsolateral |
|  |  |  |  | -3.95 | -18 | 60 | 4 |  |
|  | 0.012 | 1128 | -1.47 | -5.14 | -24 | -44 | -12 | Left fusiform |
|  |  |  |  | -4.10 | -21 | -76 | -8 |  |
|  |  |  |  | -3.94 | -32 | -44 | -2 | Left lingual gyrus |
| AN-BP vs  HC | < 0.001 | 2635 | -1.15 | -5.56 | -4 | -4 | 6 | Left thalamus, anteroventral |
|  |  |  |  | -4.37 | -14 | -10 | 14 |  |
|  |  |  |  | -3.61 | 14 | -8 | 15 | Right thalamus, ventral lateral |
|  | 0.022 | 1110 | -1.09 | -4.06 | -56 | -42 | 32 | Left supramarginal gyrus |
|  |  |  |  | -3.68 | -51 | -45 | 22 |  |
|  |  |  |  | -3.56 | -45 | -34 | 24 |  |
|  | 0.039 | 967 | -1.39 | -5.21 | 8 | -74 | 45 | Right precuneus |
|  |  |  |  | -4.73 | 26 | -66 | 62 | Right superior parietal lobule |
|  |  |  |  | -4.67 | 16 | -74 | 56 |  |

AN-R, restricting AN; AN-BP, binge-eating and purging subtype of AN.

## Table S2. Whole-brain voxel-wise analyses of cortical thickness (CT) identified brain clusters exhibiting significant differences between groups.

|  | Cluster level | | | Peak level | Coordinates, MNI (mm) | | | Anatomical location  (Desikan-Killiany atlas) |
| --- | --- | --- | --- | --- | --- | --- | --- | --- |
|  | *p FWE* | kE | Cohen's *d* | T | x | y | z |  |
| ***CT*** | | | | | | | | |
| ED vs HC | < 0.001 | 929 | -0.53 | -5.04 | -43 | 80 | 37 | Left rostral middle frontal gyrus |
|  |  |  |  | -4.85 | -27 | 80 | 26 |  |
|  |  |  |  | -4.32 | -42 | 63 | 35 |  |
|  | < 0.001 | 449 | -0.48 | -5.19 | -12 | 20 | 68 | Left paracentral lobule |
|  |  |  |  | -3.79 | -9 | 33 | 61 |  |
|  | < 0.001 | 343 | -0.52 | -4.04 | -20 | -17 | 30 | Left lingual gyrus/precuneus |
|  |  |  |  | -3.87 | -21 | -34 | 39 |  |
|  | 0.047 | 140 | -0.45 | -4.09 | -68 | 6 | 19 | Left middle temporal gyrus |
|  |  |  |  | -3.26 | -61 | -5 | 30 |  |
| AN vs HC | < 0.001 | 764 | -0.68 | -4.33 | -36 | 50 | 19 | Left rostral middle frontal gyrus |
|  |  |  |  | -4.30 | -43 | 39 | 21 |  |
|  |  |  |  | -4.24 | -46 | 22 | 20 |  |
|  | < 0.001 | 605 | -0.62 | -4.25 | -43 | -51 | 15 | Left inferior parietal lobule |
|  |  |  |  | -4.08 | -38 | -58 | 20 |  |
|  |  |  |  | -3.93 | -51 | -46 | 3 | Left banks of the superior temporal sulcus |
|  | < 0.001 | 411 | -0.53 | -4.58 | -10 | -23 | 48 | Left paracentral lobule |
|  | 0.004 | 250 | -0.47 | -4.18 | 46 | -33 | 44 | Right supramarginal gyrus |
|  |  |  |  | -4.08 | 44 | -26 | 41 |  |
|  | 0.034 | 157 | -0.59 | -4.21 | 10 | -55 | 39 | Right precuneus |
| AN vs BN | 0.048 | 142 | -0.38 | -4.64 | 10 | -54 | 41 | Right precuneus |
| ***CT, controlled for BMI*** | | | | | | | | |
| ED vs HC | < 0.001 | 630 | -0.46 | -4.36 | -43 | 80 | 37 | Left rostral middle frontal gyrus |
|  |  |  |  | -4.08 | -29 | 77 | 29 |  |
|  |  |  |  | -3.88 | -42 | 65 | 36 |  |
|  | 0.004 | 240 | -0.32 | -3.82 | -21 | -34 | 39 | Left precuneus |
|  |  |  |  | -3.52 | -14 | -10 | 25 |  |
|  |  |  |  | -3.43 | -19 | -19 | 31 |  |
| AN vs HC | < 0.001 | 941 | -0.95 | -5.58 | -47 | 21 | 20 | Left rostral middle frontal gyrus |
|  |  |  |  | -4.92 | -45 | 31 | 27 |  |
|  |  |  |  | -4.22 | -47 | 35 | 6 |  |
|  | < 0.001 | 791 | -0.83 | -4.42 | -31 | 10 | 55 | Left superior frontal gyrus |
|  |  |  |  | -4.18 | -16 | 11 | 61 |  |
|  |  |  |  | -3.78 | -9 | 29 | 57 |  |
|  | < 0.001 | 639 | -1.08 | -4.17 | -42 | -67 | 12 | Left inferior parietal lobule |
|  |  |  |  | -3.97 | -53 | -63 | -3 |  |
|  |  |  |  | -3.95 | -39 | -61 | 21 |  |
|  | < 0.001 | 362 | -0.80 | -4.29 | 27 | -61 | 46 | Right superior parietal lobule |
|  |  |  |  | -3.69 | 17 | -64 | 54 |  |
|  | < 0.001 | 335 | -0.80 | -4.04 | 28 | 26 | 40 | Right superior frontal gyrus |
|  |  |  |  | -4.01 | 13 | 49 | 39 |  |
|  | 0.001 | 290 | -0.66 | -4.09 | -21 | 40 | -17 | Left superior temporal gyrus |
|  |  |  |  | -3.68 | -33 | 23 | -22 |  |
|  |  |  |  | -3.51 | -38 | 26 | -9 |  |
| AN vs HC | 0.001 | 285 | -0.79 | -4.31 | 50 | -72 | 7 | Right lateral occipital gyrus |
|  |  |  |  | -3.91 | 54 | -62 | 4 |  |
|  | 0.027 | 165 | -0.79 | -4.43 | 52 | -6 | -30 | Right middle temporal gyrus |
|  |  |  |  | -3.95 | 44 | -10 | -42 |  |
|  | 0.033 | 157 | -0.80 | -3.95 | 44 | -27 | 44 | Right precentral gyrus |
|  | 0.039 | 149 | -0.73 | -4.19 | 25 | -68 | 6 | Right fusiform gyrus |
| AN-R vs HC | < 0.001 | 872 | -0.78 | -5.76 | -43 | -79 | 17 | Left inferior parietal lobule |
|  |  |  |  | -4.44 | -25 | -98 | -16 |  |
|  |  |  |  | -4.42 | -44 | -64 | 12 |  |
|  | < 0.001 | 711 | -0.65 | -5.35 | -45 | 1 | 30 | Left postcentral gyrus |
|  |  |  |  | -5.21 | -35 | 16 | 27 |  |
|  |  |  |  | -4.74 | -48 | 24 | 19 |  |
|  | < 0.001 | 430 | -0.85 | -4.46 | -33 | 9 | 57 | Left caudal middle frontal gyrus |
|  |  |  |  | -4.19 | -21 | 14 | 54 |  |
|  |  |  |  | -4.04 | -14 | 25 | 55 |  |
|  | < 0.001 | 379 | -0.55 | -4.28 | -33 | 21 | -15 | Left lateral orbitofrontal gyrus |
|  |  |  |  | -4.21 | -39 | 11 | -7 |  |
| AN-BP vs HC | < 0.001 | 765 | -0.89 | -4.90 | -46 | 29 | 28 | Left rostral middle frontal gyrus |
|  |  |  |  | -4.66 | -47 | 23 | 20 |  |
|  |  |  |  | -3.92 | -55 | 14 | 13 |  |
|  | < 0.001 | 705 | -0.68 | -4.54 | -62 | -40 | -6 | Left middle temporal gyrus |
|  |  |  |  | -4.25 | -42 | -67 | 12 |  |
|  |  |  |  | -3.82 | -51 | -61 | 2 |  |
|  | < 0.001 | 460 | -1.00 | -4.42 | -11 | 0 | 70 | Left superior frontal gyrus |
|  |  |  |  | -4.18 | -13 | 28 | 52 |  |
|  |  |  |  | -3.90 | -18 | 16 | 59 |  |
|  | < 0.001 | 348 | -0.72 | -3.87 | -35 | -76 | 43 | Left inferior parietal lobule |
|  |  |  |  | -3.58 | -28 | -66 | 35 |  |
|  | 0.001 | 318 | -0.89 | -4.43 | 9 | 53 | 37 | Right superior frontal gyrus |
|  |  |  |  | -3.55 | 22 | 29 | 36 |  |
|  | 0.002 | 290 | -0.75 | -3.87 | 7 | -14 | 49 | Right paracentral gyrus |
|  |  |  |  | -3.64 | 7 | 4 | 49 |  |
|  |  |  |  | -3.49 | 6 | 18 | 49 |  |
|  | 0.007 | 232 | -0.79 | -4.65 | -16 | -75 | 4 | Left precuneus |
|  | 0.047 | 147 | -0.74 | -4.58 | 24 | -64 | 7 | Right precuneus |
|  |  |  |  | -3.32 | 12 | -64 | 11 |  |
|  | 0.047 | 147 | -0.77 | -3.84 | -31 | 9 | 56 | Left pars opercularis |
|  |  |  |  | -3.71 | -43 | 14 | 48 |  |

AN-R, restricting AN; AN-BP, binge-eating and purging subtype of AN.

## Table S3. Sensitivity analyses for structural alterations.

| Group comparisons | Brain regions | Analyses using the whole sample | | |  | | | Sensitivity analyses in sub-samples | | | | | | | | | | | |
| --- | --- | --- | --- | --- | --- | --- | --- | --- | --- | --- | --- | --- | --- | --- | --- | --- | --- | --- | --- |
|  |  |  |  |  | Excluding participants aged under 22 years or above 28  years | | | Excluding HCs with a BMI over 25 or below 18.5 | | | Excluding participants with AUD diagnosis | | | Analysing participants scanned on different scanners separately | | | | | |
|  |  |  |  |  |  |  |  |  |  |  |  |  |  | GE scanner | | | Siemens scanner | | |
|  |  | Cohen's *d* | *t* | *p* | Cohen's *d* | *t* | *p* | Cohen's *d* | *t* | *p* | Cohen's *d* | *t* | *p* | Cohen's *d* | *t* | *p* | Hedge's *g* | *t* | *p* |
| ***GMV*** | | | | | | | |  | | | | | | | | | | | |
| ED vs HC | Bilateral supplementary motor area | -0.67 | -4.66 | 6.26E-06 | -0.89 | -4.80 | 4.99E-06 | -0.87 | -4.91 | 2.64E-06 | -0.52 | -3.20 | 1.69E-03 | -0.49 | -2.96 | 3.70E-03 | -0.77 | -1.53 | 1.32E-01 |
|  | Right middle frontal gyrus/ inferior frontal gyrus, pars orbitalis | -0.70 | -5.15 | 6.97E-07 | -0.97 | -5.77 | 7.38E-08 | -0.80 | -4.75 | 5.28E-06 | -0.64 | -3.91 | 1.37E-04 | -0.60 | -3.61 | 4.45E-04 | -0.76 | -1.58 | 1.20E-01 |
|  | Left thalamus | -0.55 | -4.03 | 8.23E-05 | -0.67 | -3.90 | 1.68E-04 | -0.58 | -3.45 | 7.52E-04 | -0.69 | -4.07 | 7.62E-05 | -0.75 | -4.52 | 1.43E-05 | -0.21 | -0.37 | 7.15E-01 |
|  | Left inferior frontal gyrus, pars orbitalis/ posterior orbital gyrus | -0.63 | -4.94 | 1.88E-06 | -0.87 | -5.27 | 7.05E-07 | -0.63 | -4.00 | 1.07E-04 | -0.55 | -3.30 | 1.19E-03 | -0.55 | -3.27 | 1.39E-03 | -1.16 | -2.18 | 3.43E-02 |
| AN vs HC | Bilateral supplementary motor area | -0.89 | -5.41 | 3.47E-07 | -1.08 | -5.35 | 8.80E-07 | -0.94 | -5.00 | 2.70E-06 | -0.88 | -5.34 | 5.07E-07 | -0.84 | -4.91 | 4.03E-06 | -1.04 | -1.83 | 0.08 |
|  | Left thalamus | -0.77 | -4.92 | 2.91E-06 | -0.99 | -4.88 | 5.51E-06 | -0.72 | -3.97 | 1.41E-04 | -0.78 | -4.83 | 4.54E-06 | -0.84 | -4.54 | 1.77E-05 | -0.86 | -1.40 | 0.18 |
|  | Left medial superior frontal gyrus/ pregenual anterior cingulate cortex | -0.81 | -5.12 | 1.23E-06 | -1.04 | -5.24 | 1.37E-06 | -0.89 | -4.96 | 3.21E-06 | -0.81 | -5.09 | 1.53E-06 | -0.78 | -4.70 | 9.56E-06 | -0.94 | -1.67 | 0.11 |
|  | Left inferior frontal gyrus, pars orbitalis | -0.78 | -5.26 | 6.69E-07 | -1.14 | -6.26 | 2.02E-08 | -0.74 | -4.35 | 3.50E-05 | -0.78 | -5.21 | 9.09E-07 | -0.70 | -4.44 | 2.56E-05 | -1.70 | -3.43 | 2.28E-03 |
|  | Left olfactory/ rectus/ medial orbitofrontal  cortex | -0.66 | -4.20 | 5.23E-05 | -0.85 | -3.96 | 1.65E-04 | -0.63 | -3.48 | 7.55E-04 | -0.64 | -4.08 | 8.66E-05 | -0.64 | -3.92 | 1.74E-04 | -0.91 | -1.51 | 0.14 |
|  | Right middle frontal gyrus | -0.87 | -5.29 | 6.04E-07 | -1.21 | -6.23 | 2.30E-08 | -0.88 | -4.63 | 1.19E-05 | -0.86 | -5.28 | 6.65E-07 | -0.80 | -4.57 | 1.59E-05 | -1.30 | -2.44 | 0.02 |
| AN vs BN | Left medial superior frontal gyrus/  supplementary motor  area | -0.65 | -4.38 | 2.61E-05 | -0.76 | -3.47 | 1.06E-03 | Not applicable | | | -0.61 | -3.68 | 3.83E-04 | -0.81 | -4.19 | 8.44E-05 | -0.40 | -1.78 | 0.08 |
| AN vs BN | Right striatum/ pallidum | -0.62 | -4.43 | 2.17E-05 | -0.75 | -4.33 | 6.66E-05 | Not applicable | | | -0.59 | -3.75 | 3.05E-04 | -0.55 | -3.03 | 3.48E-03 | -0.77 | -3.76 | 4.76E-04 |
| ***GMV, controlled for BMI*** | | | | | | | |  | | | | | | | | | | | |
| ED vs HC | Left lateral orbitofrontal cortex/ inferior frontal  gyrus, pars orbitalis | -0.66 | -4.82 | 3.14E-06 | -0.79 | -4.55 | 1.42E-05 | -0.72 | -4.05 | 8.67E-05 | -0.60 | -3.19 | 1.75E-03 | -0.58 | -3.08 | 2.54E-03 | -1.29 | -2.40 | 2.02E-02 |
| AN vs HC | Bilateral supplementary motor area | -1.09 | -4.90 | 3.19E-06 | -1.33 | -5.52 | 4.54E-07 | -1.16 | -4.82 | 5.69E-06 | -0.97 | -3.92 | 1.55E-04 | -0.99 | -3.91 | 1.82E-04 | -2.42 | -2.78 | 1.09E-02 |
|  | Bilateral thalamus | -0.95 | -4.42 | 2.29E-05 | -1.17 | -4.72 | 1.06E-05 | -0.89 | -3.79 | 2.69E-04 | -0.93 | -3.92 | 1.57E-04 | -1.01 | -3.73 | 3.35E-04 | -3.06E-03 | -2.72E-03 | 9.98E-01 |
|  | Left inferior parietal lobule/ supramarginal gyrus | -1.11 | -5.11 | 1.33E-06 | -1.07 | -4.33 | 4.52E-05 | -1.19 | -5.11 | 1.74E-06 | -1.10 | -4.46 | 1.98E-05 | -1.04 | -4.12 | 8.42E-05 | -2.09 | -2.13 | 4.44E-02 |
|  | Right medial superior frontal gyrus | -0.97 | -4.39 | 2.52E-05 | -1.08 | -4.20 | 7.12E-05 | -0.90 | -3.80 | 2.63E-04 | -0.99 | -4.04 | 9.82E-05 | -1.03 | -4.20 | 6.28E-05 | -0.84 | -0.89 | 3.81E-01 |
| ***CT*** | | | | | | | | | | | | | | | | | | | |
| ED vs HC | Left rostral middle frontal gyrus | -0.53 | -3.33 | 1.05E-03 | -0.77 | -3.67 | 3.73E-04 | -0.68 | -3.57 | 5.02E-04 | -0.60 | -3.51 | 5.93E-04 | -0.61 | -3.49 | 6.65E-04 | -0.87 | -1.47 | 1.49E-01 |
|  | Left paracentral lobule | -0.48 | -2.83 | 5.27E-03 | -0.49 | -2.13 | 3.50E-02 | -0.67 | -3.32 | 1.18E-03 | -0.55 | -3.08 | 2.45E-03 | -0.51 | -2.83 | 5.43E-03 | -1.18 | -2.02 | 4.90E-02 |
|  | Left lingual gyrus/precuneus | -0.52 | -3.10 | 2.23E-03 | -0.48 | -2.12 | 3.59E-02 | -0.65 | -3.28 | 1.33E-03 | -0.62 | -3.54 | 5.26E-04 | -0.64 | -3.66 | 3.81E-04 | -0.80 | -1.34 | 1.86E-01 |
|  | Left middle temporal gyrus | -0.45 | -2.88 | 4.44E-03 | -0.44 | -2.13 | 3.50E-02 | -0.64 | -3.45 | 7.59E-04 | -0.60 | -3.45 | 7.26E-04 | -0.66 | -3.80 | 2.29E-04 | -0.49 | -0.80 | 4.26E-01 |
| AN vs HC | Left rostral middle frontal gyrus | -0.68 | -3.69 | 3.50E-04 | -1.06 | -4.19 | 7.36E-05 | -0.73 | -3.56 | 5.78E-04 | -0.68 | -3.68 | 3.60E-04 | -0.65 | -3.11 | 2.49E-03 | -1.44 | -2.45 | 0.02 |
|  | Left inferior parietal lobule | -0.62 | -3.11 | 2.34E-03 | -0.87 | -3.20 | 1.97E-03 | -0.82 | -3.77 | 2.91E-04 | -0.63 | -3.17 | 1.99E-03 | -0.54 | -2.55 | 0.01 | -1.34 | -2.21 | 0.04 |
|  | Left paracentral lobule | -0.53 | -2.64 | 9.51E-03 | -0.66 | -2.35 | 2.12E-02 | -0.65 | -2.93 | 4.24E-03 | -0.52 | -2.62 | 0.01 | -0.46 | -2.14 | 0.04 | -1.30 | -2.21 | 0.04 |
|  | Right supramarginal gyrus | -0.47 | -2.29 | 2.39E-02 | -0.68 | -2.44 | 1.68E-02 | -0.49 | -2.12 | 3.68E-02 | -0.46 | -2.26 | 0.03 | -0.37 | -1.69 | 0.09 | -1.42 | -2.36 | 0.03 |
|  | Right precuneus | -0.59 | -2.94 | 3.97E-03 | -0.96 | -3.56 | 6.33E-04 | -0.74 | -3.31 | 1.35E-03 | -0.60 | -3.01 | 3.20E-03 | -0.50 | -2.36 | 0.02 | -1.23 | -1.99 | 0.06 |
| AN vs BN | Right precuneus | -0.38 | -2.06 | 4.12E-02 | -0.39 | -1.48 | 1.46E-01 | Not applicable | | | -0.31 | -1.49 | 0.14 | -0.39 | -1.62 | 0.11 | -0.40 | -1.44 | 0.16 |
| ***CT, controlled for BMI*** | | | | | | | | | | | | | | | | | | | |
| ED vs HC | Left rostral middle frontal gyrus | -0.46 | -2.62 | 9.64E-03 | -0.71 | -3.21 | 1.75E-03 | -0.49 | -2.24 | 2.65E-02 | -0.59 | -3.24 | 1.47E-03 | -0.44 | -2.21 | 2.88E-02 | -1.17 | -1.99 | 5.23E-02 |
|  | Left precuneus | -0.32 | -1.69 | 0.09 | -0.33 | -1.34 | 0.18 | -0.39 | -1.64 | 0.10 | -0.20 | -1.00 | 0.32 | -0.29 | -1.42 | 0.16 | -0.96 | -1.69 | 0.10 |
| AN vs HC | Left rostral middle frontal gyrus | -0.95 | -3.88 | 1.73E-04 | -1.37 | -4.67 | 1.24E-05 | -1.06 | -4.17 | 6.71E-05 | -0.73 | -2.65 | 9.27E-03 | -0.80 | -2.63 | 1.01E-02 | -0.87 | -0.90 | 0.38 |
|  | Right superior frontal gyrus | -0.83 | -3.12 | 2.29E-03 | -1.25 | -4.00 | 1.44E-04 | -0.85 | -3.02 | 3.26E-03 | -0.71 | -2.39 | 1.85E-02 | -0.70 | -2.23 | 2.81E-02 | -1.30 | -1.31 | 0.20 |
|  | Left inferior parietal lobule | -1.08 | -4.14 | 6.57E-05 | -1.41 | -4.62 | 1.54E-05 | -0.98 | -3.59 | 5.23E-04 | -1.09 | -3.77 | 2.60E-04 | -1.03 | -3.44 | 8.89E-04 | -1.51 | -1.52 | 0.14 |
|  | Right superior parietal lobule | -0.80 | -3.13 | 2.23E-03 | -1.00 | -3.21 | 1.97E-03 | -0.84 | -3.10 | 2.56E-03 | -0.74 | -2.60 | 0.01 | -0.71 | -2.31 | 2.33E-02 | -0.85 | -0.84 | 0.41 |
|  | Left superior temporal gyrus | -0.66 | -2.45 | 1.56E-02 | -0.86 | -2.65 | 9.64E-03 | -0.70 | -2.47 | 1.55E-02 | -0.59 | -2.00 | 0.04 | -0.59 | -1.88 | 6.40E-02 | -0.04 | -0.04 | 0.97 |
|  | Right lateral occipital gyrus | -0.79 | -2.92 | 4.25E-03 | -0.84 | -2.57 | 1.20E-02 | -0.87 | -3.05 | 2.95E-03 | -0.71 | -2.37 | 0.02 | -0.67 | -2.16 | 3.31E-02 | -0.81 | -0.77 | 0.45 |
|  | Right middle temporal gyrus | -0.79 | -2.95 | 3.83E-03 | -0.81 | -2.49 | 1.50E-02 | -0.98 | -3.51 | 6.95E-04 | -0.50 | -1.69 | 0.09 | -0.42 | -1.33 | 0.19 | -1.42 | -1.38 | 0.18 |
|  | Right precentral gyrus | -0.80 | -2.98 | 3.47E-03 | -0.94 | -2.91 | 4.75E-03 | -0.96 | -3.45 | 8.49E-04 | -0.66 | -2.19 | 0.03 | -0.61 | -1.95 | 0.05 | -0.94 | -0.91 | 0.37 |
|  | Right fusiform gyrus | -0.73 | -2.72 | 7.59E-03 | -0.60 | -1.86 | 6.64E-02 | -0.86 | -3.06 | 2.93E-03 | -0.64 | -2.11 | 0.04 | -0.65 | -2.09 | 3.90E-02 | -0.54 | -0.51 | 0.62 |

## Table S4. Relationships between neuroanatomical correlates of EDs (i.e., GMV and CT) and eating behaviours, comorbid symptoms & personality: whole sample analysis.

| Group comparisons | ROIs | Statistics | Eating behaviors | | | Comorbid symptoms | | Personality | | |
| --- | --- | --- | --- | --- | --- | --- | --- | --- | --- | --- |
|  |  |  | CR | EE | UE | Depression | Anxiety | Neuroticism | Hopelessness | Impulsivity |
| ***GMV, controlled for BMI*** | | |  | | |  | |  |  |  |
| ED < HC | Left lateral OFC & inferior frontal gyrus | *r* | -0.050 | -0.111 | -0.099 | -0.061 | -0.075 | -0.069 | -0.013 | -0.240 |
|  |  | *p* | 0.509 | 0.146 | 0.193 | 0.423 | 0.325 | 0.366 | 0.864 | 0.001^a^ |
| AN < HC | Bilateral supplementary motor area | *r* | -0.255 | 0.099 | 0.078 | -0.102 | -0.020 | -0.078 | -0.153 | 0.020 |
|  |  | *p* | 6.76E-04^a^ | 0.194 | 0.308 | 0.180 | 0.789 | 0.309 | 0.043 | 0.789 |
|  | Bilateral thalamus | *r* | -0.261 | 0.083 | 0.150 | -0.143 | -0.089 | -0.032 | -0.077 | -0.001 |
|  |  | *p* | 5.05E-04^a^ | 0.278 | 0.049 | 0.060 | 0.241 | 0.676 | 0.309 | 0.993 |
|  | Left inferior parietal lobule & left supramarginal gyrus | *r* | -0.169 | 0.008 | -0.006 | -0.035 | 0.005 | 0.080 | -0.004 | -0.111 |
|  |  | *p* | 0.026 | 0.916 | 0.937 | 0.642 | 0.951 | 0.295 | 0.960 | 0.145 |
|  | Right medial superior frontal gyrus | *r* | -0.148 | -0.005 | -0.013 | -0.079 | 0.040 | 0.022 | -0.097 | 0.007 |
|  |  | *p* | 0.052 | 0.944 | 0.869 | 0.299 | 0.600 | 0.770 | 0.201 | 0.930 |
| ***CT, controlled for BMI*** | | |  | | |  | |  |  |  |
| ED < HC | Left rostral middle frontal gyrus | *r* | -0.257 | 0.089 | 0.082 | -0.066 | -0.106 | -0.014 | -0.067 | 0.035 |
|  |  | *p* | 5.86E-04^a^ | 0.242 | 0.28 | 0.386 | 0.162 | 0.852 | 0.379 | 0.648 |
|  | Left precuneus | *r* | -0.175 | 0.030 | -0.039 | -0.067 | -0.061 | -0.029 | -0.060 | -0.013 |
|  |  | *p* | 0.020 | 0.692 | 0.605 | 0.376 | 0.426 | 0.707 | 0.432 | 0.868 |
| AN < HC (in  addition to the left rostral  MFG  identified in  the ED vs HC comparison) | Right superior frontal gyrus | *r* | -0.151 | 0.042 | 0.024 | -0.025 | -0.060 | 0.032 | -0.014 | 0.039 |
|  |  | *p* | 0.046 | 0.577 | 0.748 | 0.744 | 0.434 | 0.674 | 0.852 | 0.609 |
|  | Left inferior parietal lobule | *r* | -0.215 | -0.005 | 0.021 | -0.068 | -0.065 | -0.033 | -0.059 | 0.018 |
|  |  | *p* | 4.34E-03 | 0.946 | 0.788 | 0.372 | 0.392 | 0.666 | 0.438 | 0.816 |
|  | Right superior parietal lobule | *r* | -0.188 | 0.056 | 0.013 | -0.024 | -0.024 | -0.011 | 0.014 | 0.087 |
|  |  | *p* | 0.013 | 0.465 | 0.867 | 0.756 | 0.756 | 0.888 | 0.857 | 0.250 |
|  | Left superior temporal gyrus | *r* | -0.160 | 0.076 | 0.053 | -0.016 | -0.097 | -0.017 | -0.041 | 0.063 |
|  |  | *p* | 0.035 | 0.316 | 0.485 | 0.836 | 0.203 | 0.821 | 0.594 | 0.406 |
|  | Right lateral occipital gyrus | *r* | -0.152 | 0.013 | 0.015 | -0.018 | -0.031 | 0.044 | 0.055 | 0.032 |
|  |  | *p* | 0.045 | 0.866 | 0.849 | 0.813 | 0.682 | 0.562 | 0.473 | 0.673 |
|  | Right middle temporal gyrus | *r* | -0.076 | 0.042 | 0.016 | -0.119 | -0.093 | -0.072 | -0.040 | -0.034 |
|  |  | *p* | 0.319 | 0.584 | 0.838 | 0.117 | 0.219 | 0.346 | 0.597 | 0.652 |
| AN < HC | Right precentral gyrus | *r* | -0.103 | 0.078 | 0.015 | -0.017 | -0.056 | 0.025 | 0.008 | -0.037 |
|  |  | *p* | 0.173 | 0.307 | 0.846 | 0.822 | 0.464 | 0.744 | 0.919 | 0.626 |
|  | Right fusiform gyrus | *r* | -0.044 | -0.131 | -0.103 | -0.037 | -0.085 | -0.084 | -0.044 | -0.013 |
|  |  | *p* | 0.562 | 0.084 | 0.176 | 0.630 | 0.261 | 0.270 | 0.563 | 0.863 |

^a^ , the result is significant after Bonferroni correction.

## Table S5. Whole-brain voxel-wise analyses of fMRI tasks (the MID, EFT, and SST) identified brain clusters exhibiting significant differences in brain activations between groups.

| fMRI task and contrast | | | Cluster level | | | Peak level | Coordinates, MNI (mm) | | | Brain area (AAL3 atlas) |
| --- | --- | --- | --- | --- | --- | --- | --- | --- | --- | --- |
|  |  |  | *p_FWE_* | kE | Cohen's *d* | *T* | x | y | z |  |
| MID task | MID | ***Reward anticipation*** | | | | | | | | |
|  |  | ED vs HC | 0.002 | 58 | -0.92 | -5.69 | 30 | -82 | -50 | Right Crus II of cerebellar hemisphere |
|  |  |  |  |  |  | -3.68 | 33 | -73 | -41 |  |
|  |  |  |  |  |  | -3.52 | 39 | -73 | -47 |  |
|  |  |  | 0.005 | 51 | -0.82 | -4.29 | -12 | -85 | -44 | Left Crus II of cerebellar hemisphere |
|  |  |  |  |  |  | -3.93 | -24 | -82 | -44 |  |
|  |  |  |  |  |  | -3.51 | -3 | -82 | -41 |  |
|  |  |  | 0.007 | 48 | -0.89 | -4.41 | 0 | -76 | 4 | Left lingual gyrus |
|  |  |  |  |  |  | -3.69 | 12 | -76 | 4 | Right calcarine fissure |
|  |  |  |  |  |  | -3.37 | -9 | -76 | -14 | Left lobule VI of cerebellar hemisphere |
|  |  |  | 0.040 | 34 | -0.84 | -3.94 | 24 | 47 | 46 | Right superior frontal gyrus |
|  |  |  |  |  |  | -3.84 | 15 | 50 | 58 |  |
|  |  |  |  |  |  | -3.62 | 24 | 41 | 52 |  |
|  |  | AN vs HC | 0.003 | 57 | -1.00 | -4.02 | 12 | -79 | 7 | Right calcarine fissure |
|  |  |  |  |  |  | -3.87 | 6 | -85 | 4 |  |
|  |  |  |  |  |  | -3.74 | -3 | -76 | 1 | Left lingual gyrus |
|  |  |  | 0.015 | 41 | -0.94 | -4.10 | 36 | -76 | -14 | Right fusiform gyrus |
|  |  |  |  |  |  | -3.74 | 21 | -88 | -14 | Right lingual gyrus |
|  |  |  |  |  |  | -3.71 | 27 | -79 | -14 | Right fusiform gyrus |
|  |  |  | 0.017 | 40 | -1.06 | -4.32 | -27 | -79 | 37 | Left middle occipital gyrus |
|  |  | BN vs HC | 0.002 | 60 | -0.99 | -5.90 | 30 | -82 | -50 | Right Crus II of cerebellar hemisphere |
|  |  |  |  |  |  | -3.60 | 12 | -85 | -44 |  |
|  |  |  |  |  |  | -3.43 | 33 | -73 | -41 |  |
|  |  |  | 0.041 | 32 | -1.03 | -4.52 | -45 | -64 | -35 | Left Crus I of cerebellar hemisphere |
|  |  |  |  |  |  | -4.18 | -51 | -64 | -44 | Left Crus II of cerebellar hemisphere |
|  |  |  |  |  |  | -3.56 | -45 | -73 | -41 |  |
|  |  |  | 0.041 | 32 | -0.84 | -3.83 | 69 | -34 | -8 | Right middle temporal gyrus |
|  |  |  |  |  |  | -3.67 | 60 | -34 | -11 |  |
|  |  |  |  |  |  | -3.28 | 69 | -43 | -8 |  |
|  |  |  | 0.047 | 31 | -0.96 | -4.35 | -51 | 23 | 1 | Left inferior frontal gyrus, triangular part |
|  |  | ***Reward feedback*** | | | | | | | | |
|  |  | BN vs HC | 0.006 | 43 | -0.99 | -4.37 | 27 | -97 | 1 | Right calcarine fissure |
|  |  |  |  |  |  | -3.97 | 21 | -100 | 16 | Right superior occipital gyrus |
|  | MID anticipation and MID feedback: controlled for neuroticism, no significant results for any group comparisons | | | | | | | | | |
|  | MID, controlled for hopelessness | ***Reward anticipation*** | | | | | | | | |
|  |  | ED vs HC | 0.001 | 63 | -0.98 | -5.42 | 30 | -82 | -50 | Right Crus II of cerebellar hemisphere |
|  |  |  |  |  |  | -3.69 | 18 | -82 | -47 |  |
|  |  |  |  |  |  | -3.66 | 36 | -73 | -47 |  |
|  |  |  | 0.013 | 43 | -1.06 | -4.07 | -12 | -40 | 67 | Left precuneus |
|  |  |  |  |  |  | -4.06 | -9 | -28 | 73 | Left paracentral lobule |
|  |  |  |  |  |  | -3.38 | -27 | -22 | 76 | Left precentral gyrus |
|  |  |  | 0.027 | 37 | -1.02 | -4.13 | 12 | -22 | 73 | Right precentral gyrus |
|  |  |  |  |  |  | -3.98 | 15 | -22 | 58 | Right supplementary motor area |
|  |  | AN vs HC | 0.017 | 40 | -1.33 | -3.97 | -21 | -70 | 25 | Left superior occipital gyrus |
|  |  |  |  |  |  | -3.87 | -30 | -79 | 34 | Left middle occipital gyrus |
|  |  |  |  |  |  | -3.48 | -36 | -82 | 22 |  |
|  |  | ***Reward feedback*** | | | | | | | | |
|  |  | BN vs HC | 0.020 | 34 | -1.00 | -4.22 | -30 | -73 | 40 | Left middle occipital gyrus |
|  | MID, controlled for impulsivity | ***Reward anticipation*** | | | | | | | | |
|  |  | ED vs HC | 0.039 | 34 | -0.89 | -5.3 | 30 | -82 | -50 | Right Crus II of cerebellar hemisphere |
|  |  |  |  |  |  | -3.88 | 21 | -85 | -50 |  |
|  |  |  | 0.003 | 55 | -0.83 | -4.07 | -9 | -85 | -47 | Left Crus II of cerebellar hemisphere |
|  |  |  |  |  |  | -3.92 | -24 | -82 | -44 |  |
|  |  |  |  |  |  | -3.57 | -3 | -82 | -41 |  |
|  |  | AN vs HC | 0.005 | 50 | -1.00 | -3.93 | 12 | -79 | 7 | Right calcarine fissure |
|  |  |  |  |  |  | -3.91 | 6 | -85 | 4 |  |
|  |  |  |  |  |  | -3.73 | 0 | -76 | 4 | Left lingual gyrus |
|  |  |  | 0.021 | 38 | -1.06 | -4.26 | -27 | -79 | 37 | Left middle occipital gyrus |
|  |  |  | 0.027 | 36 | -0.93 | -4.20 | 36 | 76 | -14 | Right fusiform gyrus |
|  |  |  |  |  |  | -3.65 | 27 | -79 | -14 |  |
|  |  |  |  |  |  | -3.64 | 21 | -88 | -14 | Right lingual gyrus |
| EFT task | *EFT* | AN vs BN | < 0.001 | 74 | 0.90 | 4.65 | -33 | 11 | 7 | Left insula |
|  |  |  |  |  |  | 4.17 | -54 | -1 | -2 | Left superior temporal gyrus |
|  |  |  |  |  |  | 4.06 | -42 | 5 | 4 | Left insula |
|  | EFT, controlled for neuroticism | AN vs BN | < 0.001 | 64 | 0.90 | 4.47 | -33 | 11 | 7 | Left insula |
|  |  |  |  |  |  | 4.03 | -54 | -1 | -2 | Left superior temporal gyrus |
|  |  |  |  |  |  | 3.90 | -60 | 8 | 1 | Left rolandic operculum |
|  |  | BN vs HC | < 0.001 | 72 | -1.27 | -4.12 | 60 | -31 | 31 | Right SupraMarginal gyrus |
|  |  |  |  |  |  | -3.77 | 51 | -34 | 31 |  |
|  |  |  |  |  |  | -3.73 | 60 | -34 | 43 |  |
|  | EFT, controlled for hopelessness | AN vs BN | < 0.001 | 64 | 0.94 | 4.44 | -33 | 8 | 7 | Left insula |
|  |  |  |  |  |  | 4.09 | -45 | 5 | 4 |  |
|  |  |  |  |  |  | 3.97 | -54 | -1 | -2 | Left superior temporal gyrus |
|  | EFT, controlled for anxiety sensitivity | AN vs BN | < 0.001 | 72 | 0.91 | 4.58 | -33 | 11 | 7 | Left insula |
|  |  |  |  |  |  | 4.16 | -54 | -1 | -2 | Left superior temporal gyrus |
|  |  |  |  |  |  | 4.09 | -42 | 5 | 4 | Left insula |
|  | EFT, controlled for impulsivity | AN vs BN | 0.019 | 39 | 0.88 | 4.36 | -33 | 11 | 7 | Left insula |
|  |  |  |  |  |  | 4.31 | -42 | 5 | 4 |  |

## Table S6. Whole-brain voxel-wise analyses of fMRI tasks identified brain clusters exhibiting significant functional differences between the two AN subtypes (AN-R and AN-BP) and their differences from BN and HC groups.

| fMRI task and contrast | | Cluster level | | | Peak level | Coordinates, MNI (mm) | | | Brain area (AAL3 atlas) |
| --- | --- | --- | --- | --- | --- | --- | --- | --- | --- |
|  |  | *p FWE* | kE | Cohen's *d* | T | x | y | z |  |
| MID task | ***Reward anticipation*** | | | | | | | | |
|  | AN-R vs HC | < 0.001 | 73 | -1.37 | -4.62 | 45 | 50 | 19 | Right middle frontal gyrus |
|  |  |  |  |  | -4.14 | 39 | 53 | 31 |  |
|  |  |  |  |  | -3.77 | 33 | 53 | 43 | Right superior frontal gyrus |
|  |  | 0.015 | 38 | -1.31 | -4.88 | 3 | 38 | -8 | Right superior frontal gyrus, medial orbital |
|  |  | 0.023 | 35 | -1.27 | -4.94 | 9 | 68 | 34 | Right superior frontal gyrus, medial |
|  |  | 0.035 | 32 | -1.29 | -4.33 | -30 | -76 | 25 | Left middle occipital gyrus |
|  |  |  |  |  | -3.95 | -27 | -79 | 37 |  |
|  |  | 0.047 | 30 | -1.23 | -4.20 | 18 | -13 | 73 | Right superior frontal gyrus |
|  |  |  |  |  | -3.80 | 21 | -22 | 70 | Right precentral gyrus |
|  | ***Reward feedback*** | | | | | | | | |
|  | AN-R vs HC | 0.008 | 39 | -1.30 | -4.78 | -33 | 20 | 55 | Left middle frontal gyrus |
|  |  |  |  |  | -4.72 | -30 | 20 | 43 |  |
| EFT | AN-BP vs BN | 0.035 | 36 | 0.87 | 4.27 | -54 | -1 | -2 | Left superior temporal gyrus |
|  |  |  |  |  | 4.25 | -60 | 8 | 1 | Left Rolandic operculum |
| SST | ***Stop success*** | | | | | | | | |
|  | AN-R vs HC | 0.005 | 45 | -1.26 | 3.96 | -6 | 5 | 55 | Left supplementary motor area |
|  |  |  |  |  | 3.90 | -9 | 5 | 64 | Left supplementary motor area |
|  |  |  |  |  | 3.75 | 0 | -1 | 64 | Left supplementary motor area |
| SST | AN-R vs HC | 0.028 | 33 | -1.29 | 4.65 | 60 | 14 | 1 | Right inferior frontal gyrus, opercular part |
|  |  |  |  |  | 3.93 | 57 | 23 | -2 | Right inferior frontal gyrus, triangular part |
|  | AN-BP vs HC | 0.012 | 38 | -1.14 | -4.48 | 48 | -31 | 61 | Right postcentral gyrus |
|  |  |  |  |  | -3.76 | 42 | -22 | 64 | Right precentral gyrus |
|  |  |  |  |  | -3.63 | 54 | -22 | 58 | Right postcentral gyrus |
|  | AN-R vs AN-BP | 0.001 | 57 | -1.19 | 4.70 | 66 | -22 | 31 | Right supramarginal gyrus |
|  |  |  |  |  | 4.59 | 54 | -28 | 22 | Right Rolandic operculum |
|  |  |  |  |  | 3.35 | 63 | -28 | 19 | Right superior temporal gyrus |
|  |  | 0.013 | 38 | -1.28 | 5.18 | -51 | 23 | -14 | Left temporal pole: superior temporal gyrus |
|  |  |  |  |  | 4.06 | -57 | 17 | -5 |  |

## Table S7. Sensitivity analyses of functional alterations.

| Group comparisons | Brain regions | Analyses using the whole group | | | Sensitivity analyses in sub-samples | | | | | | | | | | | | | | |
| --- | --- | --- | --- | --- | --- | --- | --- | --- | --- | --- | --- | --- | --- | --- | --- | --- | --- | --- | --- |
|  |  |  |  |  | Excluding patients under 22 years old or above 28 years | | | Excluding HCs with a BMI over 25 or below 18.5 | | | Excluding participants with AUD diagnosis | | | Analysing participants scanned on different scanners separately | | | | | |
|  |  |  |  |  |  |  |  |  |  |  |  |  |  | GE scanner | | | Siemens scanner | | |
|  |  | Cohen's *d* | *t* | *p* | Cohen's *d* | *t* | *p* | Cohen's *d* | *t* | *p* | Cohen's *d* | *t* | *p* | Cohen's *d* | *t* | *p* | Hedge's *g* | *t* | *p* |
| ***MID task: anticipation of large reward vs no reward*** | | | | | | | | | | | | | | | | | | | |
| ED vs HC | Right Crus II of cerebellar hemisphere | -0.92 | -5.11 | 9.86E-07 | -1.18 | -4.84 | 5.20E-06 | -1.00 | -4.48 | 1.91E-05 | -0.94 | -5.16 | 8.72E-07 | -0.90 | -4.95 | 3.13E-06 | -2.09 | -4.05 | 1.88E-04 |
|  | Left Crus II of cerebellar hemisphere | -0.82 | -4.55 | 1.14E-05 | -1.06 | -4.24 | 5.33E-05 | -0.96 | -4.24 | 4.73E-05 | -0.87 | -4.71 | 6.21E-06 | -0.75 | -3.95 | 1.50E-04 | -2.22 | -4.34 | 7.47E-05 |
|  | Left lingual / right calcarine fissure/ left  Lobule VI of cerebellar hemisphere | -0.89 | -5.03 | 1.41E-06 | -1.04 | -4.15 | 7.40E-05 | -1.45 | -7.29 | 5.75E-11 | -0.90 | -4.95 | 2.18E-06 | -0.83 | -4.43 | 2.48E-05 | -1.94 | -3.64 | 6.81E-04 |
|  | Right superior frontal gyrus | -0.84 | -4.61 | 8.64E-06 | -1.20 | -4.90 | 4.06E-06 | -0.90 | -3.96 | 1.36E-04 | -0.78 | -4.15 | 5.88E-05 | -0.64 | -3.30 | 1.35E-03 | -2.57 | -5.36 | 2.49E-06 |
| AN vs HC | Right calcarine fissure/ left lingual gyrus | -1.00 | -4.59 | 1.38E-05 | -1.36 | -4.29 | 6.22E-05 | -1.37 | -6.12 | 4.21E-08 | -0.96 | -4.38 | 3.27E-05 | -0.84 | -3.46 | 9.25E-04 | -1.98 | -4.10 | 4.70E-04 |
|  | Right fusiform/ lingual gyrus | -0.94 | -4.23 | 5.49E-05 | -1.13 | -3.43 | 1.07E-03 | -1.19 | -5.10 | 2.54E-06 | -0.91 | -4.07 | 1.04E-04 | -0.54 | -4.13 | 9.88E-05 | -1.59 | -2.86 | 9.12E-03 |
|  | Left middle occipital gyrus | -1.06 | -4.99 | 2.77E-06 | -1.42 | -4.65 | 1.72E-05 | -1.14 | -4.68 | 1.30E-05 | -1.05 | -4.92 | 3.97E-06 | -0.91 | -3.80 | 3.06E-04 | -2.03 | -4.16 | 4.07E-04 |
| BN vs HC | Right Crus II of cerebellar hemisphere | -0.99 | -4.91 | 3.66E-06 | -1.39 | -4.95 | 4.43E-06 | -1.17 | -4.32 | 6.08E-05 | -1.03 | -4.93 | 4.30E-06 | -1.03 | -5.07 | 3.01E-06 | -1.54 | -2.89 | 7.93E-03 |
|  | Left Crus I/II of cerebellar hemisphere | -1.03 | -5.31 | 6.88E-07 | -1.53 | -5.77 | 1.68E-07 | -1.19 | -4.52 | 3.04E-05 | -1.05 | -5.11 | 2.05E-06 | -0.98 | -4.68 | 1.34E-05 | -1.82 | -3.54 | 1.61E-03 |
|  | Right middle temporal gyrus | -0.84 | -4.02 | 1.13E-04 | -1.31 | -4.58 | 1.83E-05 | -1.33 | -5.32 | 1.68E-06 | -0.79 | -3.53 | 6.80E-04 | -0.77 | -3.46 | 9.30E-04 | -1.65 | -3.06 | 5.18E-03 |
| BN vs HC | Left IFG, triangular part | -0.96 | -4.65 | 1.04E-05 | -1.22 | -4.14 | 9.09E-05 | -1.00 | -3.55 | 7.72E-04 | -0.87 | -3.93 | 1.77E-04 | -0.89 | -4.08 | 1.16E-04 | -1.26 | -2.17 | 4.01E-02 |
| ***MID task: feedback of large reward vs no reward*** | | | | | | | | | | | | | | | | | | | |
| ED vs HC | *NS* | | | | Not applicable | | | Not applicable | | | Not applicable | | | Not applicable | | | Not applicable | | |
| BN vs HC | Right calcarine fissure/ superior occipital gyrus | -0.99 | -4.83 | 5.09E-06 | -1.39 | -4.88 | 5.90E-06 | -1.08 | -4.06 | 1.46E-04 | -1.01 | -4.58 | 1.65E-05 | -0.81 | -3.74 | 3.74E-04 | -1.72 | -3.28 | 3.05E-03 |
| ***EFT: viewing of angry faces vs control stimuli*** | | | | | | | |  | | |  | | |  | | |  | | |
| ED vs HC | *NS* | |  |  | Not applicable | | | Not applicable | | | Not applicable | | | Not applicable | | | Not applicable | | |
| AN vs BN | Left insula/superior temporal gyrus | 0.90 | 5.47 | 2.48E-07 | 0.63 | 2.47 | 1.67E-02 | Not applicable | | | 0.92 | 4.94 | 3.38E-06 | 0.86 | 3.95 | 1.84E-04 | 0.95 | 3.78 | 4.29E-04 |

## Table S8. ROIs-based analyses of fMRI BOLD signals identified through whole-brain voxel-wise analysis, and their associations with eating behaviors, personality, and comorbid symptoms in the whole sample.

| fMRI task | Contrasts | Group comparisons | ROIs |  | Eating behaviors | | | Personality | | | Comorbid symptoms | |
| --- | --- | --- | --- | --- | --- | --- | --- | --- | --- | --- | --- | --- |
|  |  |  |  |  | CR | EE | UE | Neuroticism | Hopelessness | Impulsivity | Depression | Anxiety |
| MID | Anticipation | ED<HC | Right Crus II of cerebellar hemisphere | *r* | -0.212 | 0.027 | 0.055 | -0.324 | -0.130 | -0.142 | -0.248 | -0.171 |
|  |  |  |  | *p* | 0.010 | 0.746 | 0.503 | 5.78E-05^a^ | 0.115 | 0.086 | 2.37E-03 | 0.038 |
|  |  |  | Left Crus II of cerebellar hemisphere | *r* | -0.180 | 0.045 | 0.044 | -0.282 | -0.162 | -0.079 | -0.184 | -0.169 |
|  |  |  |  | *p* | 0.029 | 0.589 | 0.594 | 5.10E-04^a^ | 0.050 | 0.340 | 0.025 | 0.040 |
|  |  |  | Left lingual / right calcarine fissure/ left Lobule VI of  cerebellar hemisphere | *r* | -0.294 | -0.014 | 0.009 | -0.338 | -0.232 | -0.200 | -0.194 | -0.139 |
|  |  |  |  | *p* | 2.90E-04^a^ | 0.865 | 0.918 | 2.71E-05^a^ | 4.64E-03 | 0.015 | 0.018 | 0.091 |
|  |  |  | Right superior frontal gyrus | *r* | -0.190 | -0.025 | 0.037 | -0.300 | -0.154 | -0.113 | -0.105 | -0.129 |
|  |  |  |  | *p* | 0.021 | 0.766 | 0.657 | 2.12E-04^a^ | 0.062 | 0.172 | 0.203 | 0.117 |
|  |  | AN<HC | Right calcarine fissure/ left lingual gyrus | *r* | -0.326 | 0.023 | 0.043 | -0.330 | -0.290 | -0.100 | -0.228 | -0.139 |
|  |  |  |  | *p* | 5.20E-05^a^ | 0.782 | 0.606 | 3.60E-05^a^ | 2.85E-04^a^ | 0.245 | 0.006 | 0.099 |
|  |  |  | Right fusiform/ lingual gyrus | *r* | -0.310 | 0.047 | 0.101 | -0.340 | -0.230 | -0.110 | -0.206 | -0.197 |
|  |  |  |  | *p* | 1.24E-04^a^ | 0.570 | 0.222 | 3.20E-05^a^ | 0.005 | 0.174 | 0.014 | 0.019 |
|  |  |  | Left middle occipital gyrus | *r* | -0.254 | 0.045 | 0.101 | -0.240 | -0.190 | -0.080 | -0.164 | -0.235 |
|  |  |  |  | *p* | 1.81E-03^a^ | 0.588 | 0.706 | 0.004 | 0.022 | 0.314 | 0.051 | 0.005 |
|  |  | BN<HC | Right Crus II of cerebellar hemisphere | *r* | -0.200 | 0.029 | 0.054 | -0.300 | -0.100 | -0.160 | -0.223 | -0.142 |
|  |  |  |  | *p* | 0.015 | 0.729 | 0.517 | 2.53E-04^a^ | 0.021 | 0.055 | 0.008 | 0.093 |
|  |  |  | Left Crus I/II of cerebellar hemisphere | *r* | -0.211 | -0.029 | 0.008 | -0.340 | -0.130 | -0.150 | -0.212 | -0.233 |
|  |  |  |  | *p* | 0.010 | 0.726 | 0.921 | 2.70E-05^a^ | 0.115 | 0.067 | 0.011 | 0.005 |
|  |  |  | Right middle temporal gyrus | *r* | -0.078 | -0.003 | -0.004 | -0.160 | -0.050 | -0.180 | -0.086 | -0.173 |
|  |  |  |  | *p* | 0.344 | 0.974 | 0.959 | 0.054 | 0.512 | 0.028 | 0.306 | 0.039 |
|  |  |  | Left IFG, triangular part | *r* | -0.105 | -0.055 | -0.120 | -0.180 | -0.040 | -0.300 | -0.109 | -0.076 |
|  |  |  |  | *p* | 0.203 | 0.510 | 0.147 | 0.034 | 0.606 | 2.35E-04^a^ | 0.196 | 0.370 |
|  | Feedback | BN<HC | Right calcarine fissure/ superior occipital gyrus | *r* | -0.024 | -0.103 | -0.219 | -0.170 | -0.120 | -0.290 | -0.245 | -0.105 |
|  |  |  |  | *p* | 0.769 | 0.212 | 0.007 | 0.039 | 0.141 | 3.63E-04^a^ | 3.27E-03^a^ | 0.212 |
| EFT | Angry faces vs control | AN>BN | Left insula/superior temporal gyrus | *r* | 0.065 | -0.213 | -0.162 | -0.032 | 0.054 | -0.040 | -0.008 | -0.089 |
|  |  |  |  | *p* | 0.393 | 0.005 | 0.032 | 0.670 | 0.479 | 0.561 | 0.918 | 0.254 |

CR, cognitive restraint; EE, emotional eating; UE, uncontrolled eating. Models were adjusted for age and recruitment sites. ^a^, significant p-values after Bonferroni correction for multiple testing of fMRI analyses.

## Table S9. Correlations between GMVs/CT of regions that differed between groups and the brain activations in the regions identified from whole-brain fMRI analysis.

| Brain activations during reward anticipation  (ROIs from whole brain analyses)   Brain structure  (ROIs from whole brain analyses, not controlled for BMI) | | |  | ED < HC | | | | AN < HC | | |
| --- | --- | --- | --- | --- | --- | --- | --- | --- | --- | --- |
|  |  |  |  | Right Crus II of cerebellum | Left Crus II of cerebellum | Left lingual gyrus/ Right calcarine fissure | Right superior frontal gyrus | Right calcarine fissure /Left lingual gyrus | Right fusiform | Left middle occipital gyrus |
| GMV | ED < HC | Bilateral SMA + right MFG/IFGorb + left thalamus + left IFGorb | *r* | 0.139 | 0.095 | 0.170 | 0.169 |  |  |  |
|  |  |  | *p* | 0.099 | 0.262 | 0.044 | 0.046 |  |  |  |
|  | AN < HC | Bilateral SMA + left thalamus + left SFGmedial + left IFGorb + left olfactory + right MFG | *r* |  |  |  |  | 0.114 | 0.200 | 0.279 |
|  |  |  | *p* |  |  |  |  | 0.274 | 0.053 | 0.006 |
| CT | ED < HC | Left rostral MFG + left paracentral lobule + left lingual gyrus + left MTG | *r* | -0.019 | 0.028 | 0.062 | 0.067 |  |  |  |
|  |  |  | *p* | 0.826 | 0.741 | 0.466 | 0.428 |  |  |  |
|  | AN < HC | Left rostral MFG + left IPL + left paracentral + right SMG + right precuneus | *r* |  |  |  |  | 0.098 | 0.102 | 0.109 |
|  |  |  | *p* |  |  |  |  | 0.244 | 0.228 | 0.197 |

SMA, supplementary motor area; MFG, middle frontal gyrus; IFGorb, inferior frontal gyrus, pars orbitalis; SFGmedial, medial superior frontal gyrus; MFG, middle frontal gyrus; MTG, middle temporal gyrus; IPL, inferior parietal lobule; SMG, supramarginal gyrus.

## Table S10. Differences in intelligence and cognitive function between groups.

| Measures | HC | AN | BN | *F* | *p* | Post hoc analyses (Bonferroni correction) | | | | | |
| --- | --- | --- | --- | --- | --- | --- | --- | --- | --- | --- | --- |
|  |  |  |  |  |  | AN vs HC | | BN vs HC | | AN vs BN | |
|  | Mean (SD) | Mean (SD) | Mean (SD) |  |  | *p* | 95% CI | *p* | 95% CI | *p* | 95% CI |
| ***Intelligence: Weschler Adult Intelligence Scale (WAIS)*** | | | | | | | | | | | |
| Available N | 56 | 62 | 59 |  |  |  |  |  |  |  |  |
| IQ score (the average of verbal comprehension index and perceptual reasoning index scores) | 110.87 (10.25) | 107.83 (10.09) | 109.26 (19.16) | 0.68 | 0.510 |  |  |  |  |  |  |
| ***Neurocognitive tests: Cambridge Neuropsychological Test Automated Battery (CANTAB)*** | | | | | | | | | | | |
| Available N | 57 | 65 | 61 |  |  |  |  |  |  |  |  |
| Cambridge Gambling Task (CGT) | | | |  |  |  | | | | | |
| CGT Delay aversion | 0.14 (0.15) | 0.27 (0.17) | 0.25 (0.20) | 6.42 | 0.002 | 0.003 | 0.03–0.20 | 0.013 | 0.02–0.18 | 1.000 | -0.06–0.09 |
| CGT Deliberation time | 1362.19 (275.23) | 1534.95 (311.48) | 1569.71 (382.03) | 4.83 | 0.009 | 0.037 | 7.32–315.63 | 0.011 | 32.99–342.71 | 1.000 | -168.94–116.19 |
| CGT Overall proportion bet | 0.45 (0.11) | 0.52 (0.13) | 0.51 (0.13) | 6.20 | 0.002 | 0.004 | 0.02–0.14 | 0.012 | 0.01–0.13 | 1.000 | -0.05–0.06 |
| CGT Quality of decision making | 0.98 (0.04) | 0.96 (0.09) | 0.95 (0.08) | 2.79 | 0.064 |  |  |  |  |  |  |
| CGT Risk adjustment | 2.11 (0.83) | 1.45 (0.98) | 1.61 (0.96) | 8.97 | < 0.001 | < 0.001 | -1.18–-0.31 | 0.005 | -1.02–-0.14 | 0.949 | -0.57–0.24 |
| CGT Risk taking | 0.49 (0.11) | 0.56 (0.13) | 0.55 (0.13) | 5.18 | 0.006 | 0.009 | 0.01–0.13 | 0.024 | 0.01–0.13 | 1.000 | -0.05–0.06 |
| Intra-Extra Dimensional Set Shift (IED) | | | |  |  |  | | | | | |
| IED Total trials | 71.09 (12.10) | 82.08 (20.55) | 79.20 (18.61) | 6.51 | 0.002 | 0.002 | 3.68–20.29 | 0.019 | 1.21–17.90 | 1.000 | -5.25–10.12 |
| IED Total trials (adjusted) | 79.86 (48.59) | 89.77 (31.94) | 92.31 (54.71) | 2.05 | 0.132 |  |  |  |  |  |  |
| IED Completed stage trials | 65.82 (11.40) | 72.85 (17.31) | 69.36 (12.46) | 2.35 | 0.099 |  |  |  |  |  |  |
| IED Pre-ED errors | 5.74 (3.34) | 7.05 (3.40) | 7.75 (8.10) | 2.23 | 0.111 |  |  |  |  |  |  |
| IED EDS errors | 4.18 (5.32) | 7.98 (9.23) | 7.70 (10.04) | 3.96 | 0.021 | 0.034 | 0.24–8.30 | 0.049 | 0.02–8.11 | 1.000 | -3.52–3.93 |
| IED Total errors | 12.05 (7.55) | 17.49 (11.68) | 16.97 (13.46) | 4.44 | 0.013 | 0.022 | 0.64–11.25 | 0.033 | 0.35–11.01 | 1.000 | -4.65–5.17 |
| IED Total errors (adjusted) | 16.44 (26.92) | 21.34 (18.19) | 23.52 (31.56) | 1.85 | 0.161 |  |  |  |  |  |  |
| IED Completed stage errors | 9.40 (3.98) | 12.69 (7.45) | 11.57 (6.95) | 2.57 | 0.079 |  |  |  |  |  |  |
| IED Stages completed | 8.72 (1.15) | 8.66 (0.73) | 8.54 (1.13) | 1.11 | 0.333 |  |  |  |  |  |  |
| Spatial Working Memory (SWM) | | | |  |  |  | | | | | |
| SWM Between errors | 10.00 (9.06) | 24.05 (21.82) | 18.75 (16.10) | 15.54 | < 0.001 | < 0.001 | 9.95–25.42 | < 0.001 | 4.38–19.92 | 0.190 | -1.62–12.69 |
| SWM Strategy | 25.96 (5.70) | 31.26 (7.01) | 31.39 (6.25) | 13.55 | < 0.001 | < 0.001 | 2.64–8.66 | < 0.001 | 2.85–8.90 | 1.000 | -3.00–2.56 |

# References

1. Kroenke K, Spitzer RL, Williams JB (2001): The PHQ-9: validity of a brief depression severity measure. *J Gen Intern Med* 16: 606–613.

2. Saunders JB, Aasland OG, Babor TF, de la Fuente JR, Grant M (1993): Development of the Alcohol Use Disorders Identification Test (AUDIT): WHO Collaborative Project on Early Detection of Persons with Harmful Alcohol Consumption--II. *Addict Abingdon Engl* 88: 791–804.

3. World Health Organization (WHO) (1992): The ICD-10 Classification of Mental and Behavioural disorders – Clinical descriptions and diagnostic guidelines.

4. Stice E, Telch CF, Rizvi SL (2000): Development and validation of the Eating Disorder Diagnostic Scale: a brief self-report measure of anorexia, bulimia, and binge-eating disorder. *Psychol Assess* 12: 123–131.

5. American Psychiatric Association (2013): *Diagnostic and Statistical Manual of Mental Disorders*. Arlington, VA: American Psychiatric Publishing.

6. Schumann G, Loth E, Banaschewski T, Barbot A, Barker G, Büchel C, *et al.* (2010): The IMAGEN study: reinforcement-related behaviour in normal brain function and psychopathology. *Mol Psychiatry* 15: 1128–1139.

7. Sheehan DV, Lecrubier Y, Sheehan KH, Amorim P, Janavs J, Weiller E, *et al.* (1998): The Mini-International Neuropsychiatric Interview (M.I.N.I.): the development and validation of a structured diagnostic psychiatric interview for DSM-IV and ICD-10. *J Clin Psychiatry* 59 Suppl 20: 22-33;quiz 34-57.

8. Costa Jr. PT, McCrae RR (2008): The Revised NEO Personality Inventory (NEO-PI-R). *The SAGE Handbook of Personality Theory and Assessment, Vol 2: Personality Measurement and Testing*. Thousand Oaks, CA, US: Sage Publications, Inc, pp 179–198.

9. Woicik PA, Stewart SH, Pihl RO, Conrod PJ (2009): The substance use risk profile scale: A scale measuring traits linked to reinforcement-specific substance use profiles. *Addict Behav* 34: 1042–1055.

10. Anglé S, Engblom J, Eriksson T, Kautiainen S, Saha M-T, Lindfors P, *et al.* (2009): Three factor eating questionnaire-R18 as a measure of cognitive restraint, uncontrolled eating and emotional eating in a sample of young Finnish females. *Int J Behav Nutr Phys Act* 6: 41.

11. Karlsson J, Persson L-O, Sjöström L, Sullivan M (2000): Psychometric properties and factor structure of the Three-Factor Eating Questionnaire (TFEQ) in obese men and women. Results from the Swedish Obese Subjects (SOS) study. *Int J Obes* 24: 1715–1725.

12. Goodman R, Ford T, Richards H, Gatward R, Meltzer H (2000): The development and well-being assessment: description and initial validation of an integrated assessment of child and adolescent psychopathology. *J Child Psychol Psychiatry* 41: 645–655.

13. Sahakian BJ, Morris RG, Evenden JL, Heald A, Levy R, Philpot M, Robbins TW (1988): A comparative study of visuospatial memory and learning in Alzheimer-type dementia and Parkinson’s disease. *Brain J Neurol* 111 ( Pt 3): 695–718.

14. Zhang S, Wang W, Su X, Kemp GJ, Yang X, Su J, *et al.* (2018): Psychoradiological investigations of gray matter alterations in patients with anorexia nervosa. *Transl Psychiatry* 8: 1–11.

15. King JA, Geisler D, Ritschel F, Boehm I, Seidel M, Roschinski B, *et al.* (2015): Global cortical thinning in acute anorexia nervosa normalizes following long-term weight restoration. *Biol Psychiatry* 77: 624–632.

16. Walton E, Bernardoni F, Batury V-L, Bahnsen K, Larivière S, Abbate-Daga G, *et al.* (2022): Brain structure in acutely underweight and partially weight-restored Individuals with anorexia nervosa: A coordinated analysis by the ENIGMA eating disorders working group. *Biol Psychiatry* 92: 730–738.

17. Power JD, Barnes KA, Snyder AZ, Schlaggar BL, Petersen SE (2012): Spurious but systematic correlations in functional connectivity MRI networks arise from subject motion. *Neuroimage* 59: 2142–2154.

18. Bari A, Robbins TW (2013): Inhibition and impulsivity: behavioral and neural basis of response control. *Prog Neurobiol* 108: 44–79.

19. Knutson B, Fong GW, Adams CM, Varner JL, Hommer D (2001): Dissociation of reward anticipation and outcome with event-related fMRI. *Neuroreport* 12: 3683–3687.

20. Grosbras M-H, Paus T (2006): Brain networks involved in viewing angry hands or faces. *Cereb Cortex N Y N 1991* 16: 1087–1096.

21. Jia T, Ing A, Quinlan EB, Tay N, Luo Q, Francesca B, *et al.* (2020): Neurobehavioural characterisation and stratification of reinforcement-related behaviour. *Nat Hum Behav* 4: 544–558.

22. Servaas MN, van der Velde J, Costafreda SG, Horton P, Ormel J, Riese H, Aleman A (2013): Neuroticism and the brain: a quantitative meta-analysis of neuroimaging studies investigating emotion processing. *Neurosci Biobehav Rev* 37: 1518–1529.
